# Numerical investigation of cavitation and translational dynamics of 3D bubble clusters in viscoelastic tissues

**DOI:** 10.1016/j.ultsonch.2026.108125

**Published:** 2026-10-19

**Authors:** Zhenxiang Ji, Zhetao Shangguan, Jie Jin, Ruoyan Yang, Wei Li, Tianyi Yan, Dingjie Suo

**Affiliations:** School of Medical Science and Engineering, Beijing Institute of Technology, Beijing 100081, China

**Keywords:** Acoustic cavitation, Viscoelastic tissues, Bubble clusters, Collective cavitation, Translational dynamics

## Abstract

The three-dimensional organization of bubble clusters in viscoelastic tissues plays a critical role in regulating collective cavitation and translational dynamics under ultrasound excitation. To investigate how bubble topology, spatial position, and medium viscoelasticity regulate multi-bubble cavitation, a fully coupled three-dimensional framework is developed by integrating the Gilmore–Akulichev–Jeffreys model with the Peng–Robinson equation of state and an extended Doinikov translational model. Results indicate that the proposed model could describe nonlinear radial oscillation, thermodynamic response, bubble-bubble interaction, and translational motion of bubble clusters in different spatial configurations. Benchmark comparisons with representative bubble dynamics models confirm the consistency of proposed framework; compared with an isolated bubble, the three-dimensional cluster shows topology dependent collective cavitation suppression of the target-bubble response, as reflected by reduced bubble expansion, internal temperature, pressure, radial stress, and collapse strength. Under the initial-radius distribution, acoustic conditions, and material parameters examined, a transition occurs near N=15–18: high-expansion and strong-collapse responses are generally suppressed, although localized late-stage thermodynamic peaks may persist. Spatial translation further regulates cavitation activity, and an off-center position enhances the phase matching between bubble volume oscillation and the primary Bjerknes force, resulting in larger translational displacement. Parameter sweeps show that elasticity mainly shifts the collective resonance and may enhance delayed collapse, whereas viscosity strongly damps bubble dynamics and thermodynamic compression. Within the examined range, viscosities above approximately 15 mPa·s strongly attenuate inertial cavitation. These findings provide theoretical guidance for controlling bubble-mediated cavitation and translational effects in ultrasound-based applications.

## 1 Introduction

Microbubble-mediated ultrasound technologies have shown substantial clinical potential in non-invasive therapeutic applications [1], [2], including targeted drug delivery [3], [4], blood-brain barrier opening [5], [6], non-invasive tissue ablation [7], [8], sonogenetics [9], [10], and precise neuromodulation [11]. The efficacy and safety of these therapies depend fundamentally on the precise control of acoustic cavitation within biological tissues. During inertial cavitation, microbubbles could reach extreme thermodynamic states, with internal temperatures rising to several thousand Kelvin and pressures reaching hundreds of megapascals [12], thereby generating localized sonochemical and mechanical effects [13]. Accurately predicting these extreme states remains challenging because the governing dynamics involve highly nonlinear radial oscillation, non-Newtonian tissue rheology, bubble-bubble interactions, and mass transfer across the bubble interface.

To capture these complex dynamical processes, numerical models have undergone significant development. Classical formulations, such as the Gilmore and Keller–Miksis equations [14], [15], laid the groundwork for describing isolated bubble dynamics. Subsequent studies extended these models to interacting bubbles in complex media. Zilonova *et al.* investigated bubble–bubble interactions under viscoelastic drag and demonstrated that tissue elasticity and viscosity can substantially alter the phase and magnitude of inter-bubble forces [16]. Building on these interaction models, Qin *et al.* and Shen *et al.* further revealed that medium rheology, initial bubble size, and separation distance strongly regulate nonlinear oscillations, acoustic emissions, and resonance behavior in multi-bubble systems [17], [18]. Meanwhile, improvements in compressible bubble modeling, such as the Gilmore-NASG framework proposed by Denner *et al.*, have enhanced the description of extreme collapse dynamics in strongly compressible liquids [19]. For bubble migration, Klapcsik and Hegedűs compared time-resolved and time-averaged approaches for translational motion, emphasizing the importance of accurate modeling of acoustically driven bubble migration [20]. More recently, increasing attention has been directed toward collective bubble dynamics involving bubble clusters, spatial heterogeneity, and complex boundary conditions. Lesnik *et al.* demonstrated that heterogeneous bubble populations can substantially modify large-scale cavitation fields, highlighting the importance of spatial organization in collective bubble responses [21]. Zevnik *et al.* showed that interactions between collapsing bubbles and compliant structures strongly influence local mechanical effects, emphasizing the role of boundary conditions in bubble-mediated phenomena [22]. Beyond individual bubble interactions, Shen *et al.* developed a statistical bubble-cluster model and revealed that neighboring bubbles can either enhance or suppress target-bubble oscillations depending on frequency and spatial configuration [23]. These studies indicate that the cavitation response of a target bubble can be strongly regulated by neighboring bubbles, suggesting the presence of collective cavitation suppression in dense or spatially complex bubble populations. The coupling between spatial acoustic fields and bubble-cloud topology has also attracted increasing attention. Lei *et al.* demonstrated that acoustic conditions can promote mutual attraction and compact cluster formation in coupled translational bubbles under focused ultrasound excitation [24]. Chen *et al.* further revealed that the spatial organization of bubble arrays can alter the dynamic response of core bubbles through interactions with surrounding bubbles [25]. In addition, Hemmi et al. showed that polydispersity influences nonlinear acoustic-wave propagation by enhancing acoustic dispersion and energy dissipation in bubbly liquids [26]. Together, these studies emphasize that bubble configuration and acoustic coupling are critical factors governing collective cavitation behavior.

Despite these advances, existing multi-bubble models often focus on individual aspects of the problem. Some models emphasize translational motion in idealized fluids, whereas others incorporate tissue viscoelasticity without simultaneously resolving realistic gas behavior, heat and mass transfer, and three-dimensional bubble–bubble coupling. Consequently, it remains difficult to quantitatively determine how three-dimensional bubble-cluster topology induces collective cavitation suppression of a target bubble, and how this suppression is further modulated by spatial translation, medium viscoelasticity, and acoustic frequency. This problem is also relevant to recent frequency-modulated ultrasound strategies, including our previous studies on tailored dual-frequency cavitation regulation in viscoelastic media [27] and in vivo neuromodulation [28], as well as frequency chirp HIFU approaches for enhancing localized thermal and cavitation effects [29]. To address this gap, the presented study develops a fully coupled three-dimensional bubble cluster model by combining the Gilmore–Akulichev–Jeffreys formulation, the Peng–Robinson equation of state (PR EOS), and an extended three-dimensional Doinikov translational theory. This framework is applied to investigate how bubble-cluster topology, spatial translation, medium viscoelasticity, and acoustic frequency could regulate collective cavitation suppression and translational dynamics in non-Newtonian biological media. This manuscript is organized as follows: First, the proposed model is benchmarked against representative bubble-dynamics models to verify its consistency. Second, the isolated-bubble baseline is compared with two- and three-dimensional cluster configurations to evaluate topology-dependent collective cavitation suppression. Third, the effects of cluster size, spatial position, tissue elasticity, and viscosity on cavitation intensity and translational behavior are systematically examined. Finally, the influence of driving frequency is analyzed to characterize the response of 3D bubble clusters under different acoustic excitation conditions.

## 2 Methods

### 2.1 Bubble dynamics model

To accurately model gas-bubble dynamics in soft tissues, the Gilmore-Akulichev formulation was employed to characterize radial movement [30], The equation is given as:

(2.1) $$\begin{array}{c} R\ddot{R}\left(1-\frac{\dot{R}}{C}\right)+\frac{3}{2}{\dot{R}}^{2}\left(1-\frac{\dot{R}}{3C}\right)=\\ \left(1+\frac{\dot{R}}{C}\right)\left(H-\frac{3q+\left(\tau_{\mathrm{rr}}\right|R}{\rho }\right)+\frac{R}{C}\left[\dot{H}\left(1-\frac{\dot{R}}{C}\right)-\frac{3\dot{q}+\left(\tau_{\mathrm{rr}}\right|R}{\rho }\right] \end{array}$$

To describe the relationship between the bubble-bubble interaction and the viscoelasticity of the medium, we introduced Jeffreys model [16], as follows:

(2.2) $$\begin{array}{c} q+\varphi_{1}\dot{q}+\varphi_{1}\frac{\dot{R}\tau_{\mathrm{rr}}{|}_{R}}{R}=\frac{1}{3}\left[-\frac{4G}{3R^{3}}\left(R^{3}-R_{0}^{3}\right)-4\mu \frac{\dot{R}}{R}-4\mu \varphi_{2}\left(2\frac{{\dot{R}}^{2}}{R^{2}}+\frac{\ddot{R}}{R}\right)\right] \end{array}$$

(2.3) $$\begin{array}{c} \left(\tau_{\mathrm{rr}}\right|R\left(1+3\varphi_{1}\frac{\dot{R}}{R}\right)+\varphi_{1}\frac{d\left(\tau_{\mathrm{rr}}\right|R}{\mathrm{dt}}=-\frac{4G}{3}\left[1-\frac{{\left(R_{0}\right)}^{3}}{R^{3}}\right]-4\mu \frac{\dot{R}}{R}-4\mu \varphi_{2}\left(2\frac{{\dot{R}}^{2}}{R^{2}}+\frac{\ddot{R}}{R}\right) \end{array}$$

(2.4) $$\begin{array}{c} H=\frac{1}{\rho }\frac{m}{m-1}\left(p_{0}+p+B\right)\left[{\left[\frac{p_{\mathrm{in}}-\left(\frac{2\sigma }{R}\right)+\left(\tau_{\mathrm{rr}}\right|R+B}{p_{0}+p+B}\right]}^{\frac{m-1}{m}}-1\right] \end{array}$$

(2.5) $$\begin{array}{c} C=c_{0}{\left[\frac{p_{\mathrm{in}}-\left(\frac{2\sigma }{R}\right)+\left(\tau_{\mathrm{rr}}\right|R+B}{p_{0}+p+B}\right]}^{\frac{m-1}{2m}} \end{array}$$

Where *R* is the radius of the bubble, *H* is the enthalpy change of the bubble wall, ρ and $c_{0}$ are the density and the speed of sound in the medium, and m and *B* are the constants of the Gilmore equation, *G* and μ are the modulus of elasticity and viscosity in the medium, *R0* is the initial radius, the $p_{0}$ is the static background pressure, *p* is the driving sound pressure, $\varphi_{1}$ is the relaxation time of the tissue, $\varphi_{2}$ is the retardation time of the tissue, and $\tau_{\mathrm{rr}}|R$ is the radial stress at the bubble wall, and σ is the surface tension of the tissue, and $q=\int_{R}^{\infty }\frac{\tau_{\mathrm{rr}}\left(r,t\right)}{r}dr$. By systematically adjusting the $\varphi_{1}$ and $\varphi_{2}$, this generalized constitutive framework can represent different viscoelastic responses, including Zener- and Jeffreys-type behaviors.

### 2.2 PR EOS equation

The pressure within each bubble is determined by the PR EOS [27], as follows:

(2.6) $$\begin{array}{c} P_{\mathrm{in}}=\frac{R_{\mathrm{gas}}T}{V_{m}-b_{\mathrm{mix}}}-\frac{a_{\mathrm{mix}}}{V_{m}^{2}+k_{1}b_{\mathrm{mix}}V_{m}+k_{2}b_{\mathrm{mix}}^{2}} \end{array}$$

Here $V_{m}$ is the molar volume of the gas ($V_{m}=\frac{N_{A}\frac{4\pi R^{3}}{3}}{N_{t}}$, NA is Avogadro's constant, $N_{t}$ is the total number of gases), $R_{\mathrm{gas}}$ is the universal gas constant, and T is the temperature inside the bubble. It is assumed that the bubble is internally homogeneous and that bubble is assumed to remain spherical. k1 and k2 are the constants of the PR EOS. $a_{\mathrm{mix}}$ and $b_{\mathrm{mix}}$ are the coefficients of PR EOS, as follows:

(2.7) $$\begin{array}{c} a_{\mathrm{mix}}={\left(\frac{N_{air}}{N_{t}}\right)}^{2}a_{\mathrm{air}}+{\left(\frac{N_{v}}{N_{t}}\right)}^{2}a_{\mathrm{vap}}+2\sqrt{a_{\mathrm{air}}a_{\mathrm{vap}}}\left(\frac{N_{\mathrm{air}}}{N_{t}}\right)\left(\frac{N_{v}}{N_{t}}\right) \end{array}$$

Where $N_{t}$ is the sum of the number of water vapor and air molecules within the bubble, $N_{t}=N_{\mathrm{vap}}+N_{\mathrm{air}}$.

(2.8) $$\begin{array}{c} b_{\mathrm{mix}}=\left(\frac{N_{air}}{N_{t}}\right)b_{\mathrm{air}}+\left(\frac{N_{v}}{N_{t}}\right)b_{\mathrm{vap}} \end{array}$$

### 2.3 Water vapor transport

The transport of water vapor across the bubble wall was described by evaporation and condensation processes using the Hertz–Knudsen equation [31]. The molar rate of water-vapor transport at the bubble wall is calculated as:

(2.9) $$\begin{array}{c} \dot{n_{v}}=\frac{4\pi R^{2}}{M_{v}}\frac{\alpha_{m}\left(\sqrt{\frac{8R_{\mathrm{gas}}T_{s}}{\pi M_{v}}}\right)}{4}\left[\left(322\exp^{\left(b_{1}\theta^{\frac{2}{6}}+b_{2}\theta^{\frac{4}{6}}+b_{3}\theta^{\frac{8}{6}}+b_{4}\theta^{\frac{18}{6}}+b_{5}\theta^{\frac{37}{6}}+b_{6}\theta^{\frac{71}{6}}\right)}\right)-\frac{M_{v}\left(\frac{N_{v}}{N_{t}}\right)}{V}\right] \end{array}$$

where $\theta =1-\frac{T_{0}}{647.096}$, $M_{v}$ is the molar mass of water vapor, and the temperature at the bubble wall is $T_{s}$*=*$T_{0}$.

### 2.4 Gas transport

The transport of non-condensable gases across the bubble wall was described using Fick’s law together with the boundary-layer approximation [32]. The instantaneous molar rate of gas diffusion for species is given by:

(2.10) $$\begin{array}{c} {\dot{n}}_{g,i}=4\pi R^{2}D_{i}\frac{c_{\infty ,i}-c_{s,i}}{L_{g,i}} \end{array}$$

### 2.5 Heat transfer

The bubble heat transfer rate $\dot{Q}$ and the thermal boundary layer thickness $L_{\mathrm{th}}$ can be approximated as [33] :

(2.11) $$\begin{array}{c} \dot{Q}=4\pi R^{2}\lambda_{m}\frac{T_{0}-T}{L_{\mathrm{th}}} \end{array}$$

(2.12) $$\begin{array}{c} L_{\mathrm{th}}=\min\left(\sqrt{\frac{RK_{m}}{\left|\dot{R}\right|}},\frac{R}{\pi }\right) \end{array}$$

### 2.6 Bubble temperature

The first law of thermodynamics is employed for calculating the internal energy change inside the bubble [34], [13], [27]:

(2.13) $$\begin{array}{c} \dot{E}=\sum_{i=1}^{4}\left(h_{i}-u_{i}\right){\dot{N}}_{i}+\dot{Q}-\dot{W} \end{array}$$

(2.14) $$\begin{array}{c} h_{i}=\frac{f_{i}+2}{2}K_{B}T_{0} \end{array}$$

(2.15) $$\begin{array}{c} u_{i}=\left[\frac{f_{i}}{2}+\sum_{1}^{n}\frac{\frac{\theta_{n}}{T_{b}}}{\exp\left(\frac{\theta_{n}}{T_{b}}\right)-1}\right]K_{B}T_{b} \end{array}$$

The rate of temperature change inside the bubble can be found by coupling Equations(2.1), (2.4), (2.5), (2.9), (2.10), (2.13), $\dot{T}$ is as follows:

(2.16) $$\begin{array}{c} \dot{T}=\frac{\dot{E}}{C_{\nu ,mix}}=\frac{\sum_{i=1}^{4}\left(h_{i}-u_{i}\right){\dot{N}}_{i}}{C_{\nu ,mix}}+\frac{\dot{Q}}{C_{\nu ,mix}}-\frac{\dot{W}}{C_{\nu ,mix}} \end{array}$$

(2.17) $$\begin{array}{c} \dot{W}=P_{i}\dot{V} \end{array}$$

(2.18) $$\begin{array}{c} \dot{V}=4\pi R^{\dot{2}}\dot{R} \end{array}$$

(2.19) $$\begin{array}{c} C_{\nu ,mix}=K_{B}\sum_{i=1}^{4}\left(\frac{f_{i}}{2}+\sum_{l}^{n}\left(\frac{\left(\frac{\theta_{n}}{{T_{b}}^{2}\exp\left(\frac{\theta_{n}}{T_{b}}\right)}\right)}{{\left(\exp\left(\frac{\theta_{n}}{T_{b}}\right)-1\right)}^{2}}\right)\right)N_{i} \end{array}$$

Where $\dot{E}$ is the rate of change of total energy, $\dot{W}$ is the work done by the expansion of the bubble, $\dot{V}$ is the rate of change of bubble volume, the $C_{v,mix}$ is the heat capacity of an equal volume gas mixture, $N_{i}$ is the number of molecules of each gas, and n is the number of the characteristic vibrational temperatures $\theta_{n}$.

### 2.7 3D Bubble-bubble interactions and translational dynamics

The translational motion equation originally proposed by Doinikov’s theory [35], [36] was adapted to characterize the bubble interactions and extended to three dimensions. The primary Bjerknes force was also incorporated to describe the acoustic-field-induced translation of the bubbles [16], Equations (2.20), (2.21) represent the original one-dimensional two-bubble formulation and are introduced here as the theoretical basis for the subsequent three-dimensional extension:

(2.20) $$\begin{array}{c} R_{i}{\ddot{R}}_{i}\left(1-\frac{{\dot{R}}_{i}}{C_{i}}\right)+\frac{3}{2}{\dot{R}}_{i}^{2}\left(1-\frac{{\dot{R}}_{i}}{3C_{i}}\right)-\left(1+\frac{{\dot{R}}_{i}}{C_{i}}\right)\left(H_{i}-\frac{\tau_{\mathrm{rr}}^{\left(i\right)}{|}_{R}}{\rho_{t}}+\frac{3q_{i}}{\rho_{t}}\right)\\ -\frac{R_{i}}{C_{i}}\left[{\dot{H}}_{i}\left(1-\frac{{\dot{R}}_{i}}{C_{i}}\right)-\frac{1}{\rho_{t}}\frac{d}{\mathrm{dt}}\tau_{\mathrm{rr}}^{\left(i\right)}{|}_{R}+\frac{1}{\rho_{t}}\frac{d}{\mathrm{dt}}\left(3q_{i}\right)\right]\\ =\frac{{\dot{x}}_{i}^{2}}{4}-\frac{R_{j}^{2}{\ddot{R}}_{j}+2R_{j}{\dot{R}}_{j}^{2}}{d}-{\left(-1\right)}^{i}\frac{R_{j}^{2}\left({\dot{x}}_{i}{\dot{R}}_{j}+R_{j}{\ddot{x}}_{j}+5{\dot{R}}_{j}{\dot{x}}_{j}\right)}{2d^{2}}-\frac{R_{j}^{3}{\dot{x}}_{j}\left({\dot{x}}_{i}+2{\dot{x}}_{j}\right)}{2d^{3}} \end{array}$$

(2.21) $$\begin{array}{c} \frac{R_{i}{\ddot{x}}_{i}}{3}+{\dot{R}}_{i}{\dot{x}}_{i}-\frac{{\left(-1\right)}^{i}}{d^{2}}\frac{d}{\mathrm{dt}}\left(R_{i}R_{j}^{2}{\dot{R}}_{j}\right)-\frac{R_{j}^{2}}{d^{3}}\left(R_{i}R_{j}{\ddot{x}}_{j}+R_{j}{\dot{R}}_{i}{\dot{x}}_{j}+5R_{i}{\dot{R}}_{j}{\dot{x}}_{j}\right)=\frac{F_{ex}^{\left(i\right)}}{2\pi \rho_{t}R_{i}^{2}} \end{array}$$

The equation for the additional variable $q_{i}$ and the modified equation for the stress $\tau_{\mathrm{rr}}^{\left(i\right)}{|}_{R}$ should be coupled with the equation as follows:

(2.22) $$\begin{array}{c} q_{i}+\varphi_{1}{\dot{q}}_{i}+\varphi_{1}\frac{{\dot{R}}_{i}\tau_{\mathrm{rr}}^{\left(i\right)}{|}_{R}}{R_{i}}=\frac{1}{3}\left\{-\frac{4G}{3R_{i}^{3}}\left[R_{i}^{3}-{\left(R_{0}^{\left(i\right)}\right)}^{3}\right]-4\mu \frac{{\dot{R}}_{i}}{R_{i}}-4\mu \varphi_{2}\left(2\frac{{\dot{R}}_{i}^{2}}{R_{i}^{2}}+\frac{{\ddot{R}}_{i}}{R_{i}}\right)\right\} \end{array}$$

(2.23) $$\begin{array}{c} \tau_{\mathrm{rr}}^{\left(i\right)}{|}_{R}\left(1+3\varphi_{1}\frac{{\dot{R}}_{i}}{R_{i}}\right)+\varphi_{1}\frac{d\tau_{\mathrm{rr}}^{\left(i\right)}{|}_{R}}{\mathrm{dt}}=-\frac{4G}{3}\left[1-\frac{{\left(R_{0}^{\left(i\right)}\right)}^{3}}{R_{i}^{3}}\right]-4\mu \frac{{\dot{R}}_{i}}{R_{i}}-4\mu \varphi_{2}\left(2\frac{{\dot{R}}_{i}^{2}}{R_{i}^{2}}+\frac{{\ddot{R}}_{i}}{R_{i}}\right) \end{array}$$

To accurately represent the spatial distribution of the bubble cloud, the position of the i-th microbubble is denoted by the vector $X_{i}={\left[x_{i},y_{i},z_{i}\right]}^{T}$, and its translational velocity is $V_{i}=\dot{X_{i}}$. The Euclidean distance between the centers of the i-th and j-th bubbles is $d_{\mathrm{ij}}=\left|X_{i}-X_{j}\right|$, with the unit direction vector pointing from bubble i to j defined as $n_{\mathrm{ij}}=\frac{X_{j}-X_{i}}{d_{\mathrm{ij}}}$.

By extending the double bubble dynamics model, the fully coupled 3D radial governing equation for the i-th bubble in a cloud of N bubbles is formulated as:

(2.24) $$\begin{array}{c} R_{i}{\ddot{R}}_{i}\left(1-\frac{{\dot{R}}_{i}}{C_{i}}\right)+\frac{3}{2}{\dot{R}}_{i}^{2}\left(1-\frac{{\dot{R}}_{i}}{3C_{i}}\right)-\left(1+\frac{{\dot{R}}_{i}}{C_{i}}\right)\left(H_{i}-\frac{\tau_{\mathrm{rr}}^{\left(i\right)}{|}_{R}}{\rho_{t}}+\frac{3q_{i}}{\rho_{t}}\right)\\ -\frac{R_{i}}{C_{i}}\left[{\dot{H}}_{i}\left(1-\frac{{\dot{R}}_{i}}{C_{i}}\right)-\frac{1}{\rho_{t}}\frac{d}{\mathrm{dt}}\tau_{\mathrm{rr}}^{\left(i\right)}{|}_{R}+\frac{1}{\rho_{t}}\frac{d}{\mathrm{dt}}\left(3q_{i}\right)\right]\\ =\frac{|v_{i}{|}^{2}}{4}-\sum_{j=1,j\neq i}^{N}\left[\begin{array}{c} \frac{R_{j}^{2}{\ddot{R}}_{j}+2R_{j}{\dot{R}}_{j}^{2}}{d_{\mathrm{ij}}}+\frac{R_{j}^{2}}{2d_{\mathrm{ij}}^{2}}\left(v_{i}\cdot n_{\mathrm{ij}}{\dot{R}}_{j}+R_{j}{\dot{v}}_{j}\cdot n_{\mathrm{ij}}+5{\dot{R}}_{j}v_{j}\cdot n_{\mathrm{ij}}\right)\\ +\frac{R_{j}^{3}v_{j}\cdot n_{\mathrm{ij}}}{2d_{\mathrm{ij}}^{3}}\left(v_{i}\cdot n_{\mathrm{ij}}+2v_{j}\cdot n_{\mathrm{ij}}\right) \end{array}\right]\# \end{array}$$

Furthermore, the equation (2.21) also needs to be expanded as follows:

(2.25) $$\begin{array}{c} \frac{R_{i}\dot{V_{i}}}{3}+{\dot{R}}_{i}V_{i}+\sum_{j=1,j\neq i}^{N}\frac{n_{\mathrm{ij}}}{{d_{\mathrm{ij}}}^{2}}\frac{d}{\mathrm{dt}}\left(R_{i}R_{j}^{2}{\dot{R}}_{j}\right)-\sum_{j=1,j\neq i}^{N}\frac{R_{j}^{2}}{{d_{\mathrm{ij}}}^{3}}\left(R_{i}R_{j}\dot{V_{j}}+R_{j}{\dot{R}}_{i}V_{j}+5R_{i}{\dot{R}}_{j}V_{j}\right)=\frac{F_{ex}^{\left(i\right)}}{2\pi \rho_{t}R_{i}^{2}} \end{array}$$

Previous studies have investigated the interaction between bubbles in fluid media. Therefore, viscous resistance is usually expressed in the form of Levich.

(2.26) $$\begin{array}{c} F_{D}=\frac{2\pi }{3}\rho R_{B}^{3}\dot{U}+2\pi \rho U{\dot{R}}_{B}R_{B}^{2}+12\pi R_{B}\int_{-\infty }^{t}E\left(t-t_{1}\right)U\left(t_{1}\right)dt_{1} \end{array}$$

The external force in Equation (2.21) should only be equal to the viscous elastic part of the resistance and the primary Bjerknes force part, that is the third term in Equation (2.26), which can be written as (assuming the fluid is at rest at t = 0):

(2.27) $$\begin{array}{c} F_{ex}^{\left(i\right)}=-D_{ve}^{\left(i\right)}+{F_{\mathrm{pri}}}^{\left(i\right)}=-12\pi R_{i}\int_{0}^{t}E\left(t-t_{1}\right)U_{i}\left(t_{1}\right)dt_{1}+{F_{\mathrm{pri}}}^{\left(i\right)} \end{array}$$

(2.28) $$\begin{array}{c} \frac{dD_{\mathrm{ve},i}}{\mathrm{dt}}+\left(\frac{1}{\varphi_{1}}-\frac{{\dot{R}}_{i}}{R_{i}}\right)D_{\mathrm{ve},i}=12\pi R_{i}\left[\frac{G}{\varphi_{1}}\left(x_{i}-x_{0,i}\right)+\frac{\mu }{\varphi_{1}}v_{i}+\frac{\mu \varphi_{2}}{\varphi_{1}}{\dot{v}}_{i}\right] \end{array}$$

Where $D_{\mathrm{ve},i}$ denotes the viscoelastic drag variable, $v_{i}$ denotes translational velocity, and $dv_{i}/dt$ denotes translational acceleration. The last term is the acceleration contribution weighted by the retardation time $\varphi_{2}$. The displacement and velocity terms correspond to the elastic restoring force and viscous resistance, respectively. In three dimensions, Eq. (2.28) is applied to the x, y, and z components of the translational motion.

(2.29) $$\begin{array}{c} {F_{\mathrm{pri}}}^{\left(i\right)}=-\frac{4\pi }{3}R_{i}^{3}kp_{A}\sin\left(2\pi ft\right)\sin\left(kz_{i}\right) \end{array}$$

Where the wave number $k=\frac{2\pi f}{c}$.

### 2.8 Driving acoustic pressure

In this study, the effect of nonlinear wave propagation is not considered, and the driving sound pressure is as follows:

(2.30) $$\begin{array}{c} \frac{\partial^{2}p}{\partial t^{2}}-c^{2}\frac{\partial^{2}p}{\partial z^{2}}=0 \end{array}$$

The acoustic pressure field can be expressed as follows:

(2.31) $$\begin{array}{c} p\left(z,t\right)=p_{A}\sin\left(\frac{2\pi f}{c}\left(ct-z\right)\right)=p_{A}\sin\left(wt-kz\right) \end{array}$$

Here, z is the axial coordinate, c is the sound speed, and $p_{A}$ is the acoustic-pressure amplitude. The driving frequency is f, the angular frequency is ω=2πf, and the wavenumber is k=ω/c.

Equation (2.31) describes a traveling wave. In the present simulations, a standing wave at a single frequency is assumed within the framework of linear acoustics. The acoustic pressure at the position of bubble i is expressed as follows: [24]:

(2.32) $$\begin{array}{c} P_{t}\left(t\right)=p_{A}\sin\left(2\pi ft\right)cos\left(kz_{i}\right) \end{array}$$

Equation (2.32) describes the standing wave assumed in the simulations, rather than a direct simplification of Eq. (2.31). The term $cos\left(kz_{i}\right)$ accounts for the spatial variation of the acoustic pressure amplitude, where $z_{i}$ denotes the axial position of bubble i.

### 2.9 Simulation setup and model assumptions

The set of coupled ordinary differential equations was solved using the fourth-order Runge–Kutta 5(4) method. The coupled ordinary differential equations were solved using an implicit integration method with adaptive time stepping. The relative and absolute error tolerances were set to $10^{-5}$ and $10^{-8}$, respectively. The integration step size was adjusted automatically to satisfy these tolerances.

The baseline parameters used in this study are summarized in Table 1, and any modifications under specific simulation conditions are stated in the corresponding sections. Additional derivations related to the PR EOS and the internal thermal model of bubbles can be found in previous studies [13], [27]. For each three-dimensional bubble-cluster configuration, the initial coordinates of the bubbles (N) were generated using a grid-based constrained randomization method with a coordinate spacing of 50 μm. The smallest bubble was fixed at the cluster center ((0,0,0)) and defined as the target bubble, while the remaining bubbles were randomly assigned to discrete candidate coordinates in the (x), (y), and (z) directions; a fixed random seed was used to ensure reproducibility. The 50 μm spacing was chosen to approximate characteristic inter-capillary distances in soft-tissue microvascular beds [37]. The simulated array scale of N=4∼40 was selected according to reported local microbubble concentrations of 10^2^ to 10^3^ bubbles/mm^3^ after systemic dilution of commercial ultrasound contrast agents[38], which corresponds to tens of bubbles within a localized therapeutic volume. Comparable initial microbubble separations might also promote compact cluster formation under acoustic excitation through secondary Bjerknes forces[39].

**Table 1:** Baseline simulation parameters.

Parameters	Definition	Value	Unit
T0	Ambient temperature	293.15	K
P0	Static pressure	1.013 × 10^5^	Pa
*m*	Empirical constant	7	/
$\varphi_{1}$	Tissue relaxation time	3e-9	s
$\varphi_{2}$	the retardation time of the tissue	3e-10	s
*B*	Empirical constant	3.1309× 10^8^	Pa
Rg	Universal gas constant	8.314472	$J\cdot {\mathrm{mol}}^{-1}\cdot K$
$k_{B}$	Boltzmann constant	1.38065 × 10^-23^	$J\cdot K^{-1}$
ε	Accommodation coefficient	0.4	/
$M_{\mathrm{vap}}$	Molar mass of water vapor	18.015268 × 10^-3^	$\mathrm{kg}\cdot {\mathrm{mol}}^{-1}$
$M_{\mathrm{air}}$	Molar mass of air	28.97 × 10^-3^	$\mathrm{kg}\cdot {\mathrm{mol}}^{-1}$
$M_{N_{2}}$	Molar mass of nitrogen	28 × 10^-3^	$\mathrm{kg}\cdot {\mathrm{mol}}^{-1}$
$M_{O_{2}}$	Molar mass of oxygen	31.9988 × 10^-3^	$\mathrm{kg}\cdot {\mathrm{mol}}^{-1}$
$M_{A_{r}}$	Molar mass of argon	39.95 × 10^-3^	$\mathrm{kg}\cdot {\mathrm{mol}}^{-1}$
ρ	Liquid density	998	$\mathrm{Kg}/m^{3}$
c0	Sound speed used in the model	1482	m/s
σ	Surface tension	0.073	N/m

For each cluster containing N bubbles, the initial radii were equally spaced between 2 and 8 μm. The bubble with the smallest initial radius, was designated as the target bubble and initially positioned at the origin. The spatial configuration was generated from a Cartesian grid with a spacing of 50 μm using a fixed random seed of 1. Thus, randomness was introduced into the spatial arrangement, whereas the initial radii were prescribed deterministically. The expansion ratio of each bubble was defined as $R_{i}\left(t\right)/R_{i,0}$, where $R_{i,0}$ denotes its initial radius.

The presented model assumes a homogeneous viscoelastic medium and a linearly propagating acoustic field. Each bubble is assumed to remain spherical; therefore, nonspherical deformation, jet formation, shell effects, coalescence, fragmentation, and direct bubble contact are not considered. These assumptions allow the model to isolate the coupled effects of bubble-cluster topology, spatial translation, and medium viscoelasticity on collective cavitation suppression (Fig. 1).

**Fig. 1:**
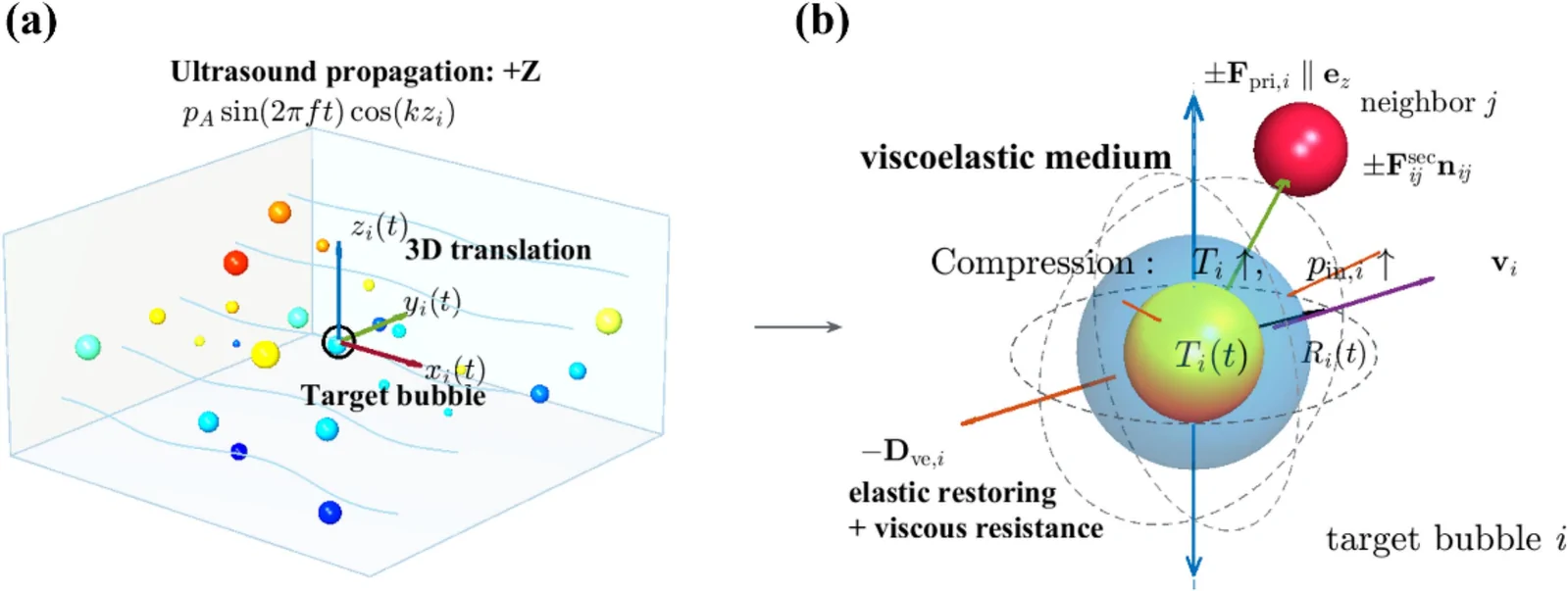
Coupled 3D framework for ultrasound-driven microbubble-cluster dynamics in a viscoelastic fluid. (a) 3D spatial distribution of the bubble cluster. The light-blue trajectories denote the imposed acoustic field $p_{A}sin\left(2\pi ft\right)cos\left(kz_{i}\right)$, while $x_{i}\left(t\right)$, $y_{i}\left(t\right)$, and $z_{i}\left(t\right)$ indicate the translational degrees of freedom of target bubble i. (b) Thermodynamic and translational-force balance for target bubble i. Compression increases the internal temperature $T_{i}\left(t\right)$ and pressure $p_{in,i}$. The instantaneous radius $R_{i}\left(t\right)$, bubble velocity $v_{i}$, primary Bjerknes force $F_{pri,i}$, viscoelastic resistance $D_{ve,i}$, and secondary interaction $F_{\mathrm{ij}}^{sec}n_{\mathrm{ij}}$ with neighboring bubble j are shown.

## 3 Results

### 3.1 Benchmark comparison and baseline response

To examine the reliability of the proposed 3D multi-bubble framework, reduced two-bubble and three-bubble cases were first benchmarked using the target-bubble expansion ratio($E_{tar}\left(t\right)$=R/R0). In this study, inertial cavitation was identified when the maximum value of $E_{tar}\left(t\right)$ during the simulation exceeded 2[40]. Maximum values exceeding 5 and 6 were used to indicate strong and very strong responses, respectively, under the conditions investigated. For the two-bubble benchmark, the present model was reduced to a planar configuration with z=0, and the target-bubble response was compared with that predicted by the viscoelastic bubble-interaction model of Zilonova *et al.*[16]. For the three-bubble case, the target bubble initially located at ((0,0,0)) was compared with the multi-bubble model of Qin *et al.*[41]. The acoustic and material parameters were selected according to recent in vivo magnetic resonance elastography and rheological measurements of soft biological tissues, ensuring physiologically relevant viscoelastic conditions [16], [17], [30], [42]. Furthermore, the GAZ-PR-EOS single-bubble model developed in our previous work[27] was used as the isolated-bubble baseline for subsequent topology-dependent comparisons.

As shown in Fig. 2(a), the reduced two-bubble form of the present model reproduces the main expansion-collapse pattern predicted by Zilonova *et al.* over five acoustic cycles. Both models show closely matched oscillation periods and collapse timings, with the maximum expansion remaining in a similar range of approximately 4. For the three-bubble case, Fig. 2(b) shows that the present model and Qin *et al.*’s model also exhibit comparable radial-oscillation trends for the target bubble, although small differences in peak expansion and rebound behavior remain because of different gas-state descriptions, thermal assumptions, and viscoelastic coupling formulations. Using the GAZPREOS single-bubble result as the isolated reference state [27], Fig. 2(c, d) further shows that the 2D and 3D configurations slightly reduce the radial expansion but markedly suppress the collapse-induced thermal response. In particular, the isolated GAZPREOS bubble reaches a peak internal temperature close to 1400K, whereas the 2D and 3D clustered cases reduce the temperature peaks to approximately 750K. These results indicate that neighboring bubbles preserve the main radial oscillation pattern while weakening the thermodynamic intensity of collapse, providing baseline evidence for topology-dependent collective cavitation suppression.

**Fig. 2:**
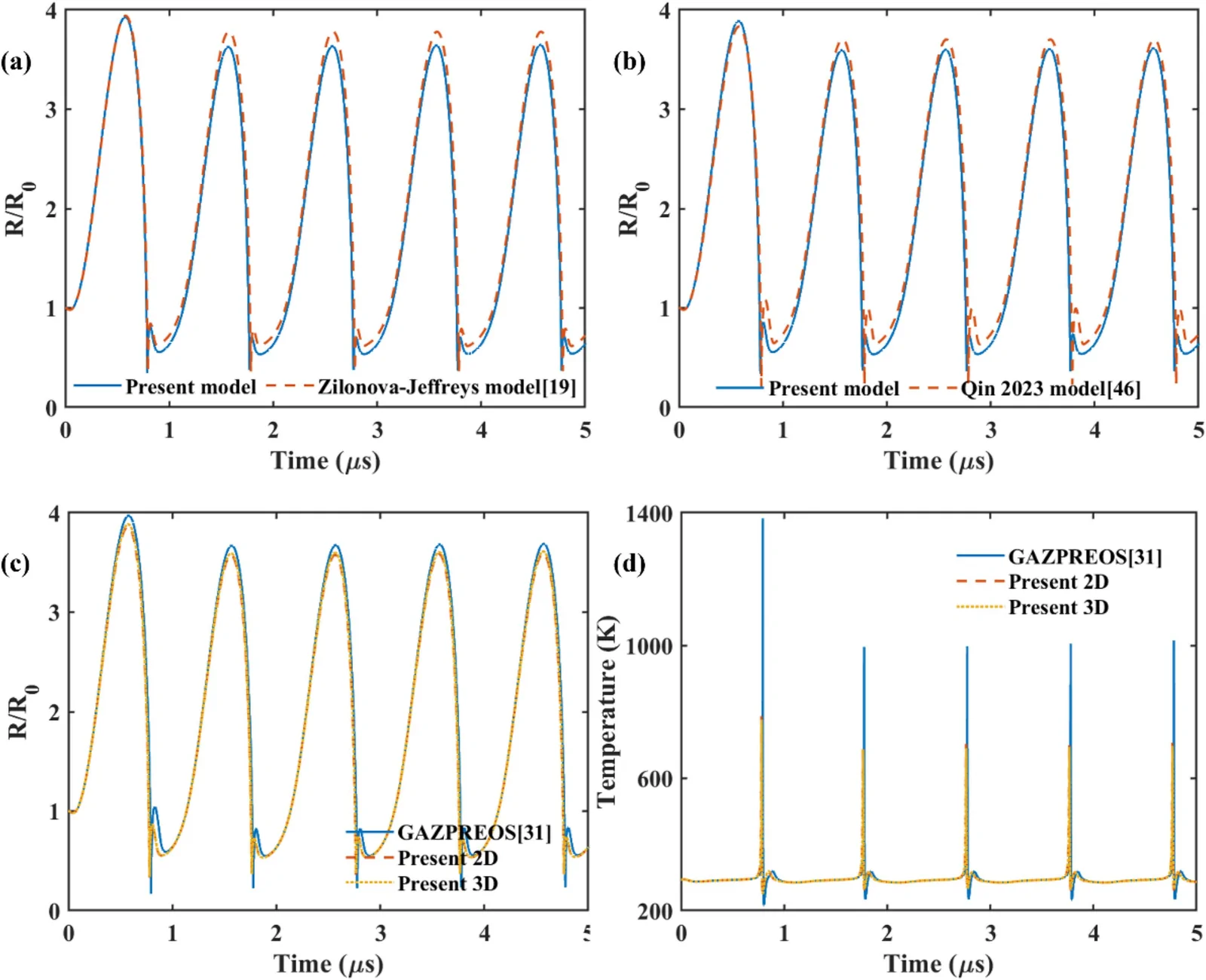
Benchmark comparison and baseline response. (a) Two-bubble radial benchmark between the present model and the model of Zilonova *et al.* (b) Three-bubble radial benchmark between the present model and the model of Qin *et al.* (c) Radial response of the isolated GAZPREOS bubble and the present 2D/3D configurations. (d) Internal temperature response of the isolated GAZPREOS bubble and the present 2D/3D configurations. The main simulation parameters were $p_{A}$ =0.6 MPa, (f=1.0) MHz, R0=1.25 μm, d=150 μm, G=10 kPa, and μ=15 mPa· s.

### 3.2 Effects of spatial translation and collective cavitation suppression

Fig. 3 describes the influence of initial axial position on the translational and cavitation responses of a 10-bubble cluster. As the cluster position shifts from Z=0 to 150 μm, the target bubble exhibits stepwise translational migration, with displacement jumps occurring near collapse instants. At (Z=0), the strongest collapse response is obtained, with peak radial stress of approximately 235 MPa, internal pressure near 1600 MPa, and temperature exceeding 3000 K. However, the total displacement remains limited to about 4.6 μm. In contrast, the intermediate position of Z=100 μm produces the largest total displacement, approximately 10 μm, indicating that translational migration is maximized when the primary Bjerknes force is better phase-matched with the bubble volume oscillation. Further translation to Z=150 μm reduces both cavitation intensity and translational response.

**Fig. 3:**
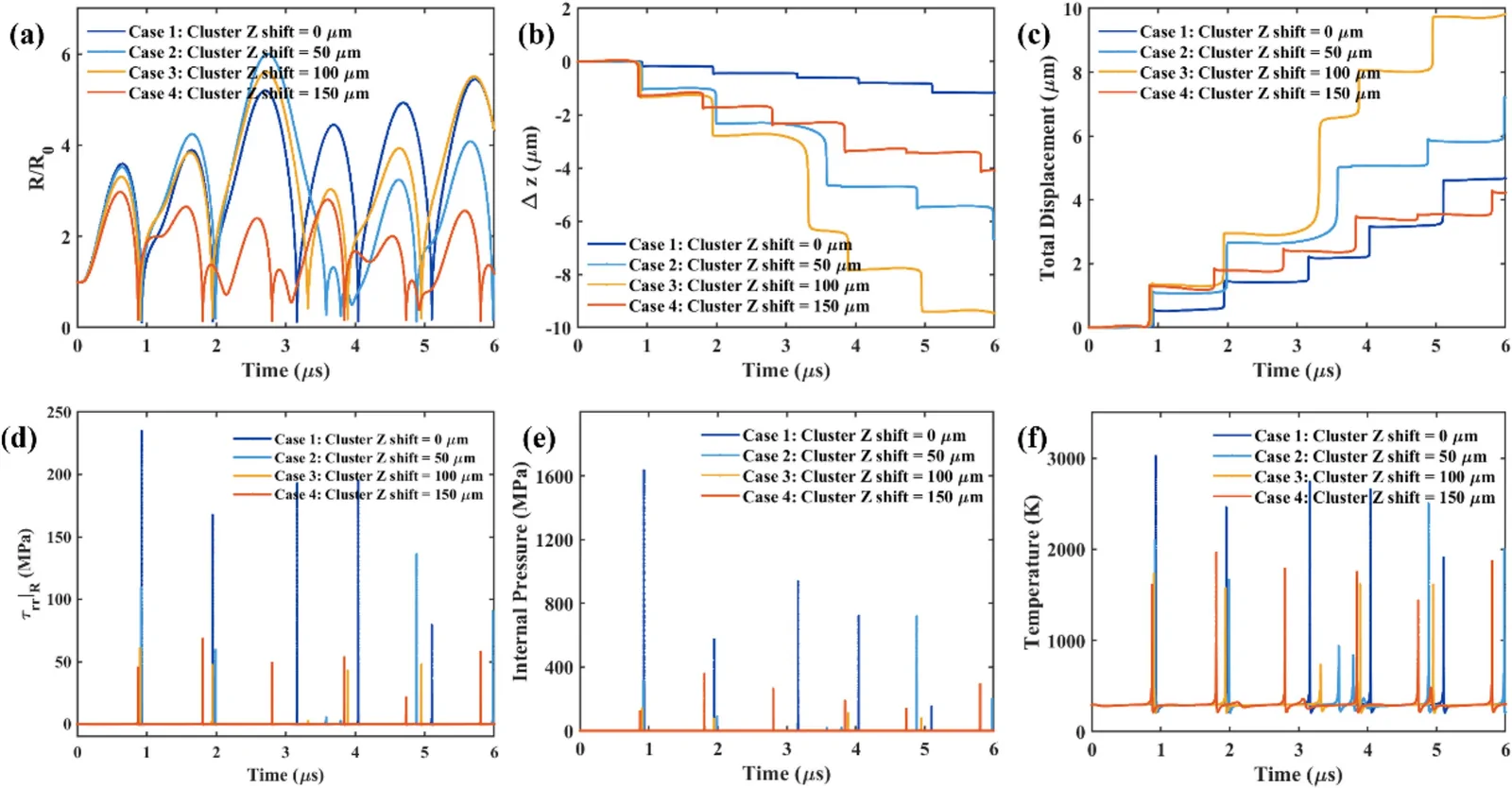
Coupled effects of spatial translation on the translational dynamics and cavitation extremes of the 3D bubble cluster. Temporal evolution is presented for (a) bubble expansion ratio, (b) axial displacement, (c) total displacement, (d) radial stress, (e) internal pressure, and (f) internal temperature. The simulation parameters were configured to represent a blood-mimicking medium characterized by a viscosity of 3 mPa·s and the elasticity is 0 Pa [40], [43]. The cluster scale is fixed at a population of 10 bubbles, driven by an acoustic pressure of 0.5 MPa.

Fig. 4 compares the effect of cluster size on the target-bubble response. Sparse clusters with N=4 or 10 bubbles mainly undergo primary-Bjerknes-force-driven translation, whereas dense clusters with N=20 or 30 exhibit altered axial motion and stronger late-stage collective responses [44]. In the dense cases, the late-cycle internal pressure and temperature can increase to approximately 4000 MPa and 3700 K, respectively.

**Fig. 4:**
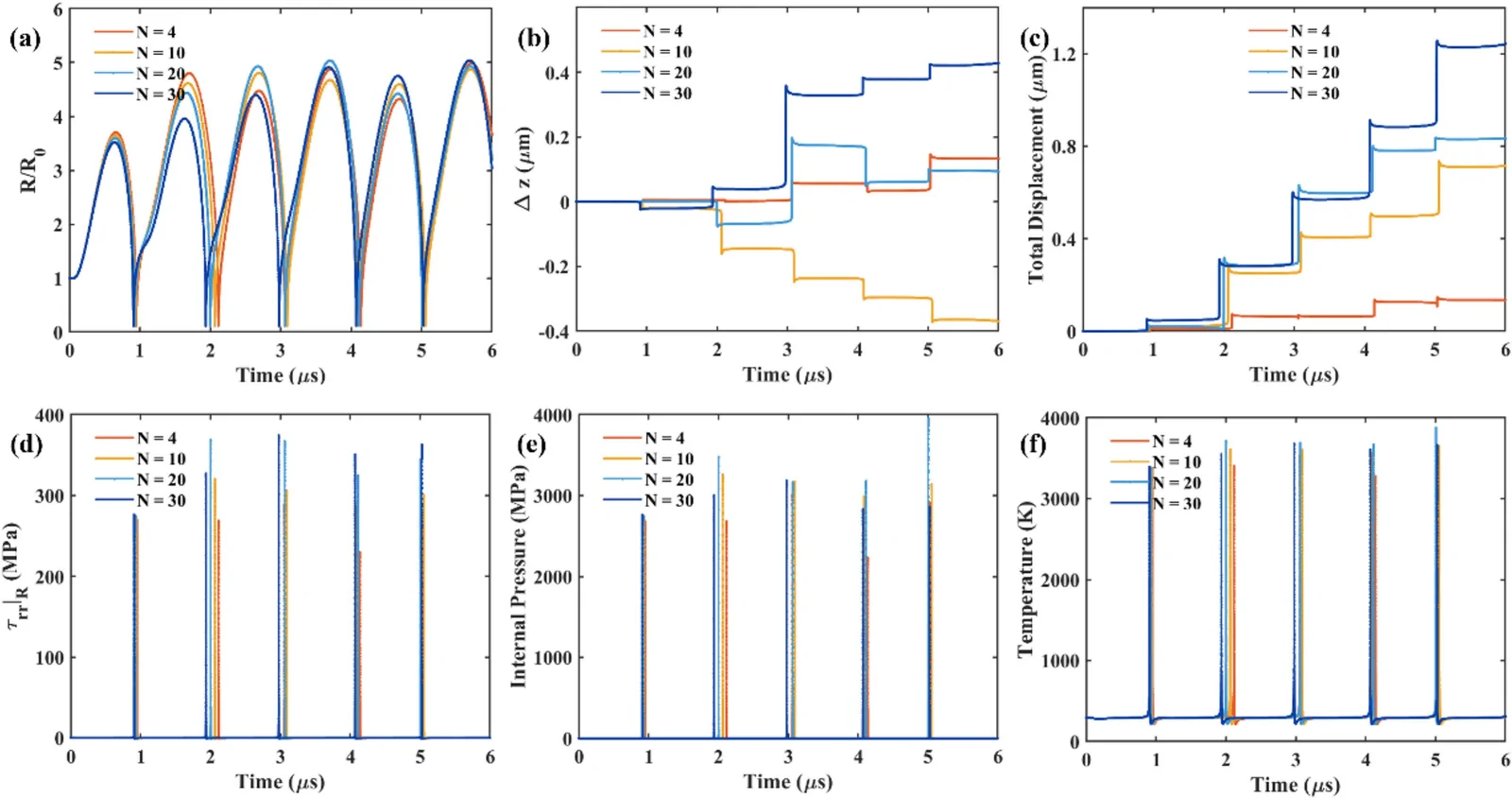
Influence of cluster topology on translational migration and localized thermodynamic extremes. Transient profiles of (a) bubble expansion ratio, (b) axial displacement, (c) total displacement, (d) radial stress, (e) internal pressure, and (f) internal temperature are mapped across varying cluster scales ranging from 4 to 30 bubbles. The target cluster is positioned precisely at the acoustic focal zone representing a Z-axis shift of 0 μm. Consistent with the previous configuration, the environmental parameters simulate blood properties with a viscosity of 3 mPa·s and an elasticity of 0 Pa under an applied acoustic pressure of 0.5 MPa.

To comprehensively map the multi-dimensional boundaries where spatial gradients and cluster topology jointly regulate the cavitation response under physiological constraints, Fig. 5 characterizes the coupled N-Z parameter space to evaluate the combined effects of cluster topology and spatial translation on cavitation dynamics. The bubble cluster exhibits inertial cavitation. Within this regime, positioning the cluster at a Z-shift between 80 and 150 μm results in a maximum translational displacement exceeding 3.5 μm, as shown in the localized region of the displacement mapping. Conversely, for N > 18, the high-response region becomes markedly restricted: peak radial stress, internal temperature, and internal pressure decrease over most evaluated Z-coordinates. These boundaries are consistent with recent observations of cavitation-induced pressure saturation and incident-wave attenuation in densely clustered arrays [45]. The transition near N=15–18 is specific to the initial-radius distribution, acoustic conditions, and material parameters examined here. Beyond this range, the region of high-expansion and strong-collapse response generally contracts, although some dense-cluster configurations retain localized late-stage thermodynamic peaks, as illustrated in Fig. 4.

**Fig. 5:**
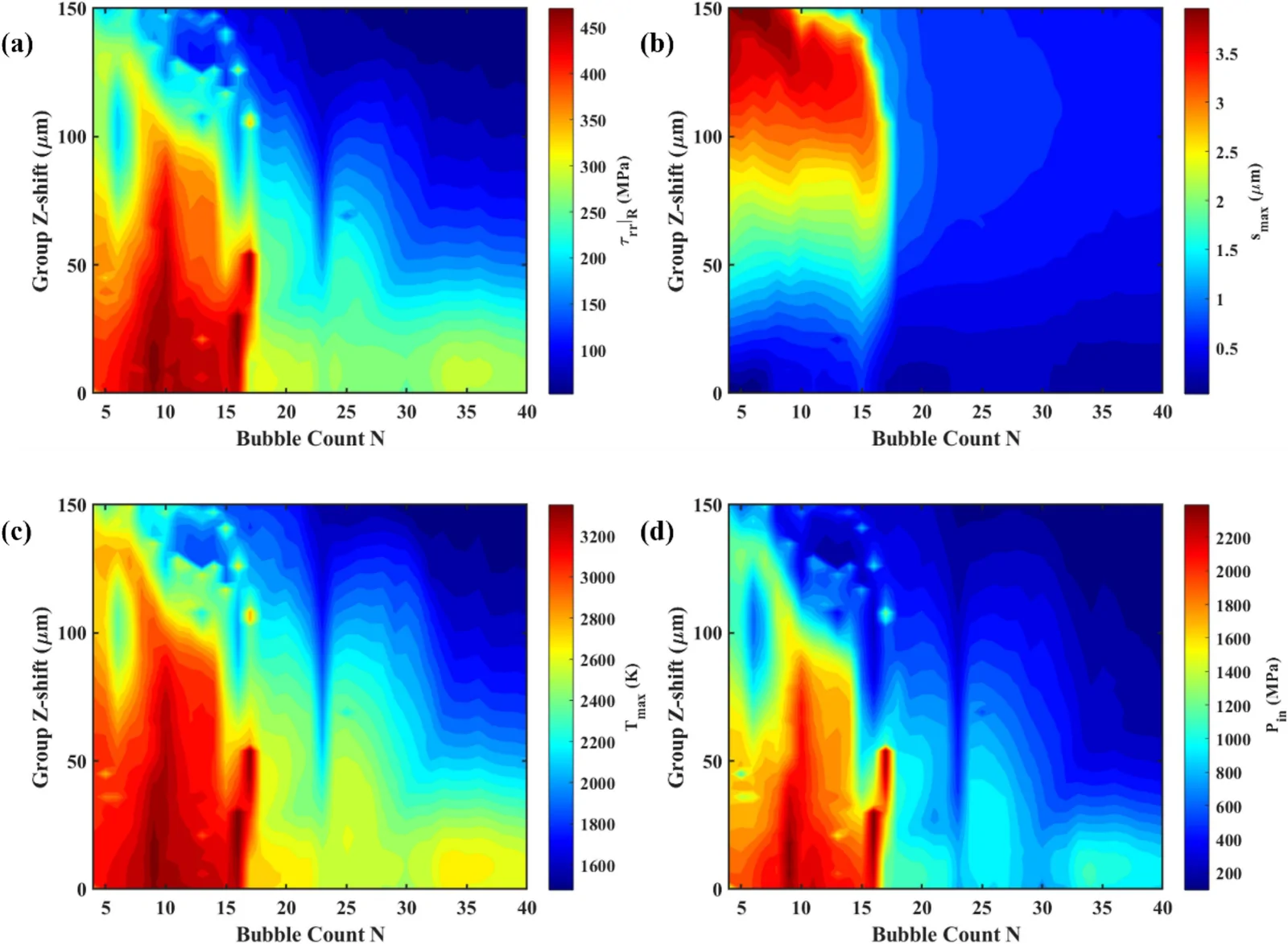
Contour mappings of the coupled effects of cluster scale and spatial translation on cavitation parameters in a viscoelastic medium. The panels display the maximum (a) radial stress, (b) translational displacement, (c) internal temperature, and (d) internal pressure. Parameters: the bubble count (N) ranges from 4 to 40, the spatial shift (Z) ranges from 0 to 150 μm, the elasticity is 1 kPa, the viscosity is 5 mPa·s, and the acoustic driving pressure is 0.5 MPa.

### 3.3 Coupled effects of medium viscoelasticity and bubble clusters

Fig. 6 evaluates the influence of tissue elasticity on the transient response of the target bubble. Under purely viscous and weakly elastic conditions, the bubble maintains strong radial oscillation and stepwise translational migration. Increasing the elasticity generally restricts radial expansion and reduces thermodynamic intensity. However, an intermediate elasticity of G=10 kPa produces a delayed collapse amplification after approximately 3 μs, where the normalized radius exceeds 6 and a sharp upward displacement spike is generated. This result indicates that tissue elasticity can shift the collapse phase and locally enhance late-stage cavitation rather than simply damping the bubble response.

**Fig. 6:**
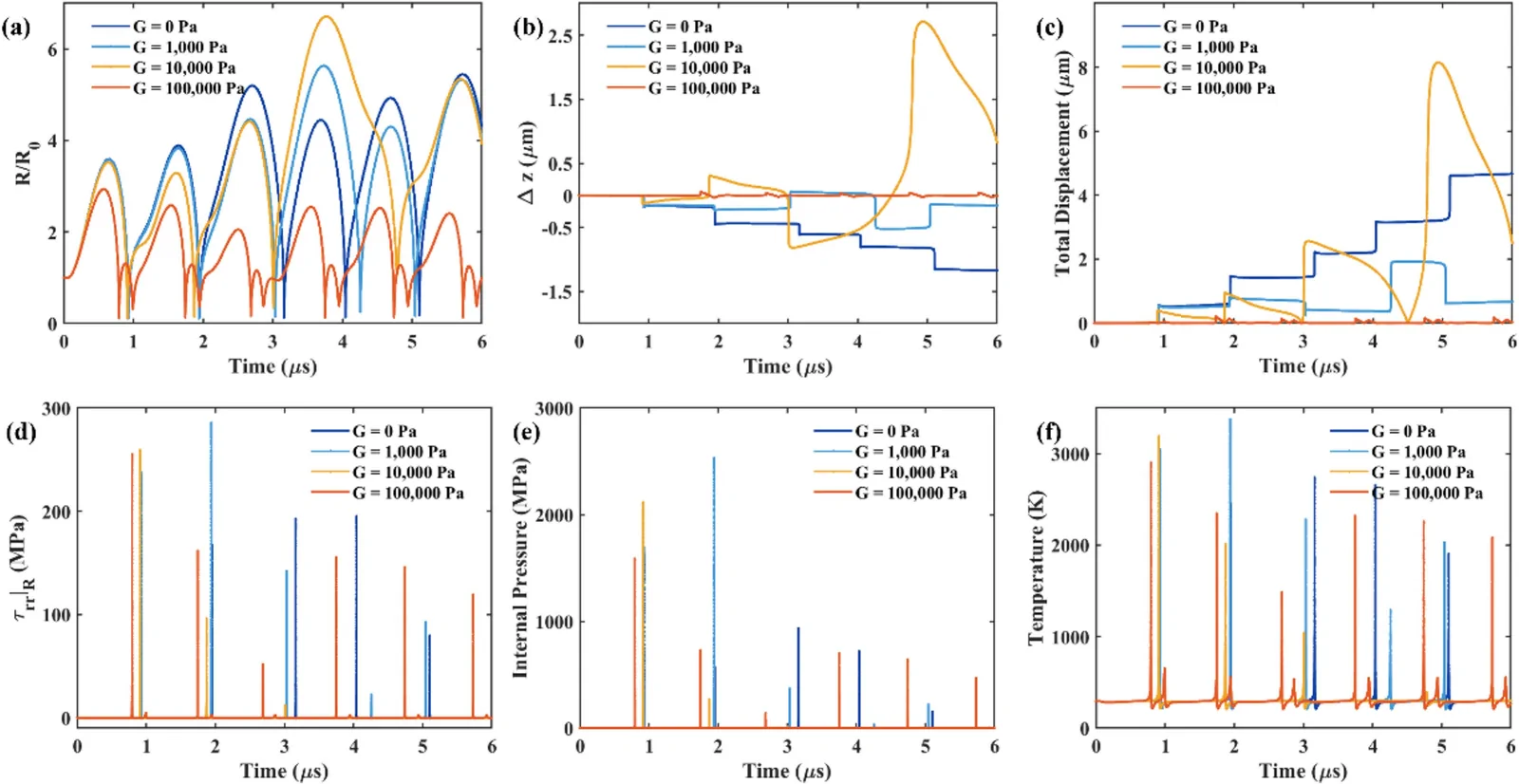
Transient dynamic profiles of the core target bubble evaluated across varying medium shear moduli of 0, 1,000, 10,000, and 100,000 Pa. The temporal evolution is plotted for (a) bubble expansion ratio, (b) axial displacement, (c) total displacement, (d) viscoelastic radial stress, (e) internal pressure, and (f) internal temperature. The cluster scale is fixed at a population of N = 10 bubbles, and the cluster is positioned precisely at the acoustic focal zone, the viscosity is 3 mPa·s, and the acoustic driving pressure is 0.5 MPa.

Fig. 7 characterizes the effect of viscosity. At μ=0.001 Pa·s, the target bubble exhibits strong expansion with approaching 7 and total displacement reaching nearly 12 μm. Increasing the viscosity to 0.005 Pa·s reduces radial expansion but still allows pronounced collapse, with radial stress near 300 MPa and internal pressure near 1300 MPa. When the viscosity increases to 0.015 and 0.050 Pa·s, radial oscillation is strongly damped, translational displacement is nearly eliminated, and thermodynamic peaks are substantially reduced.

**Fig. 7:**
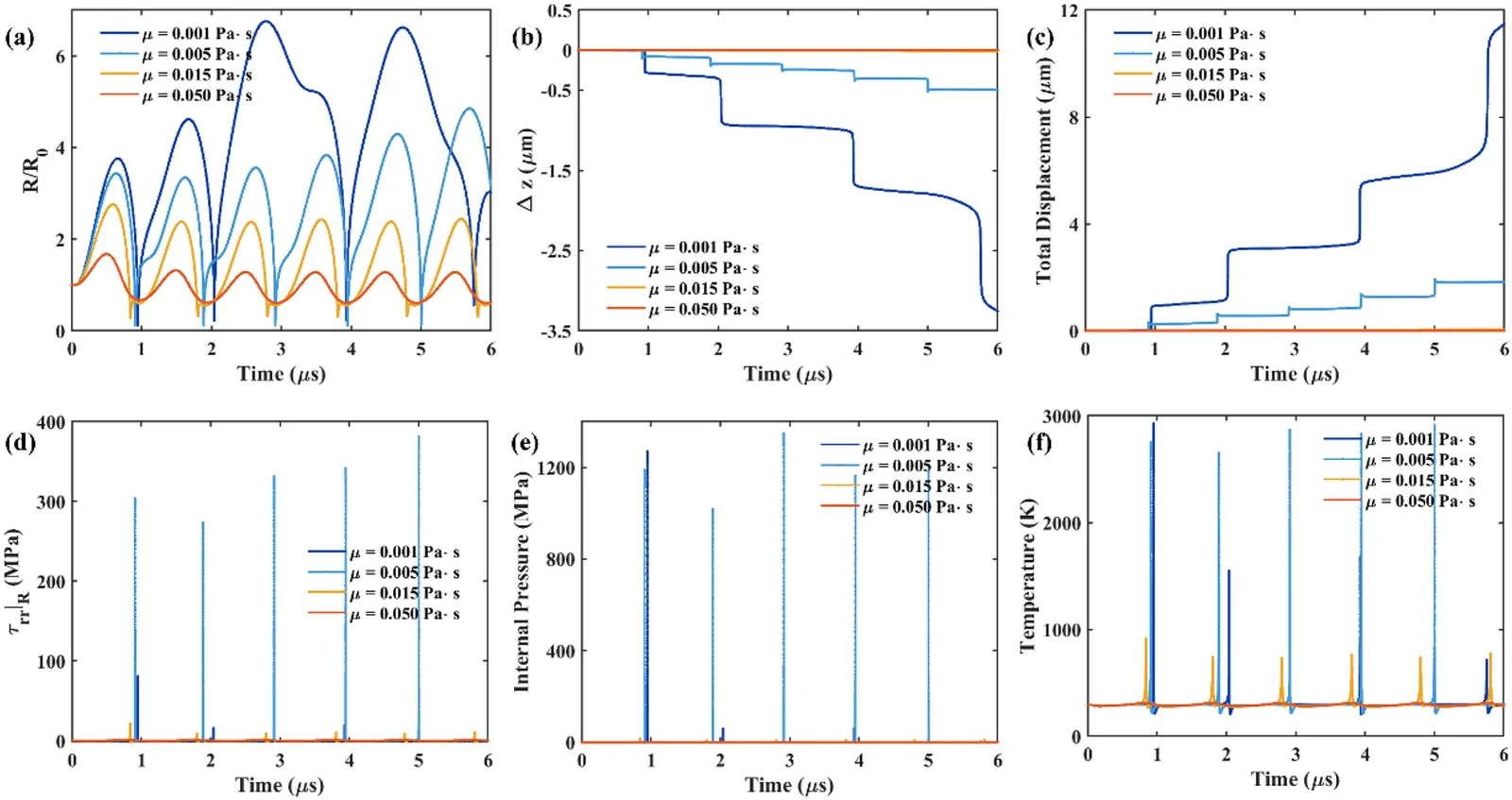
Transient dynamic profiles of the core target bubble evaluated across varying dynamic viscosities of 0.001, 0.005, 0.015, and 0.050 Pa·s. The temporal evolution is plotted for (a) bubble expansion ratio, (b) axial displacement, (c) total displacement, (d) viscoelastic radial stress, (e) internal pressure, and (f) internal temperature. The fixed parameters are set to G = 0 Pa, N = 10, Z = 0 μm, and $P_{A}$= 0.5 MPa.

To systematically evaluate the coupled influence of cluster topology and tissue elasticity, Fig. 8 evaluates the coupled influence of cluster scale and tissue elasticity across N=4∼40 and G=0∼100 kPa. Energetic inertial cavitation mainly occurs in compliant media with sparse arrays, particularly at (G<20) kPa and (N<15), where the peak radial stress exceeds 450 MPa, the internal temperature rises above 3300 K, and the internal pressure approaches 2600 MPa. As elasticity increases, the high-response region becomes progressively restricted. In contrast, the maximum translational displacement is concentrated within an intermediate elastic range of G=10–20 kPa, indicating that radial cavitation intensity and translational migration do not necessarily share the same optimal parameter region.

**Fig. 8:**
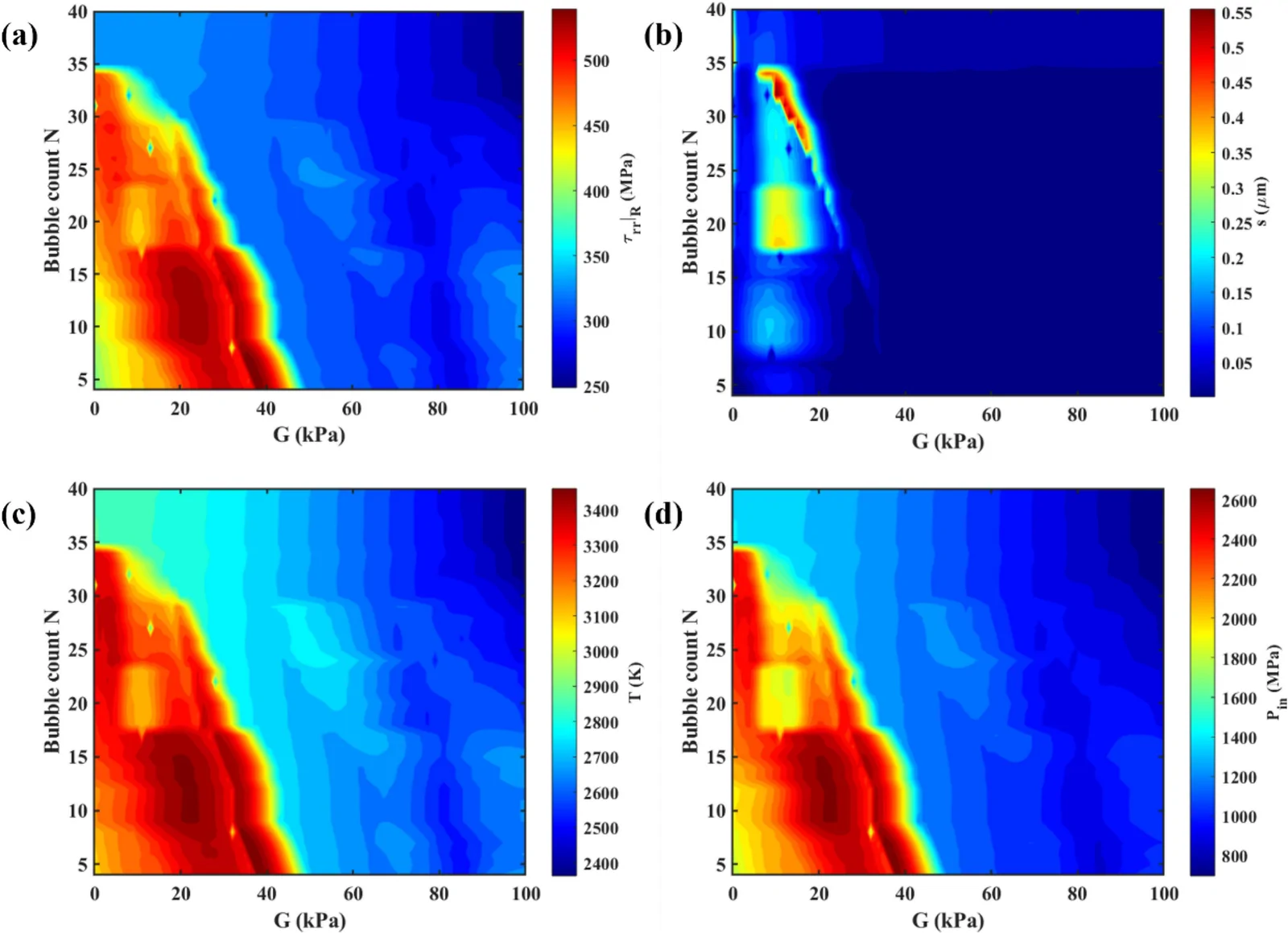
Two-dimensional contour plots illustrating the highly coupled, nonlinear effects of bubble cluster scale and tissue elasticity. The panels display the absolute maximum values for (a) viscoelastic radial stress, (b) translational displacement, (c) internal temperature, and (d) internal pressure. The fixed environmental parameters are set to a viscosity of 5 mPa·s under an acoustic driving pressure of 0.5 MPa.

To isolate the numerical influence of spatial translation under elastic constraints, Fig. 9 further evaluates the coupled effects of spatial translation and tissue elasticity at a fixed low viscosity. A critical elasticity threshold appears near G=25 kPa. When G>25 kPa, all cavitation metrics decline sharply across the evaluated spatial coordinates: radial stress drops below 150 MPa, internal pressure decreases to below 500 MPa, internal temperature falls below 2000 K, and translational displacement approaches 0 μm. In the compliant regime of G<25 kPa, spatial off-centering partially restores the dynamic response, with the largest displacement occurring around Z=50∼100 μm.

**Fig. 9:**
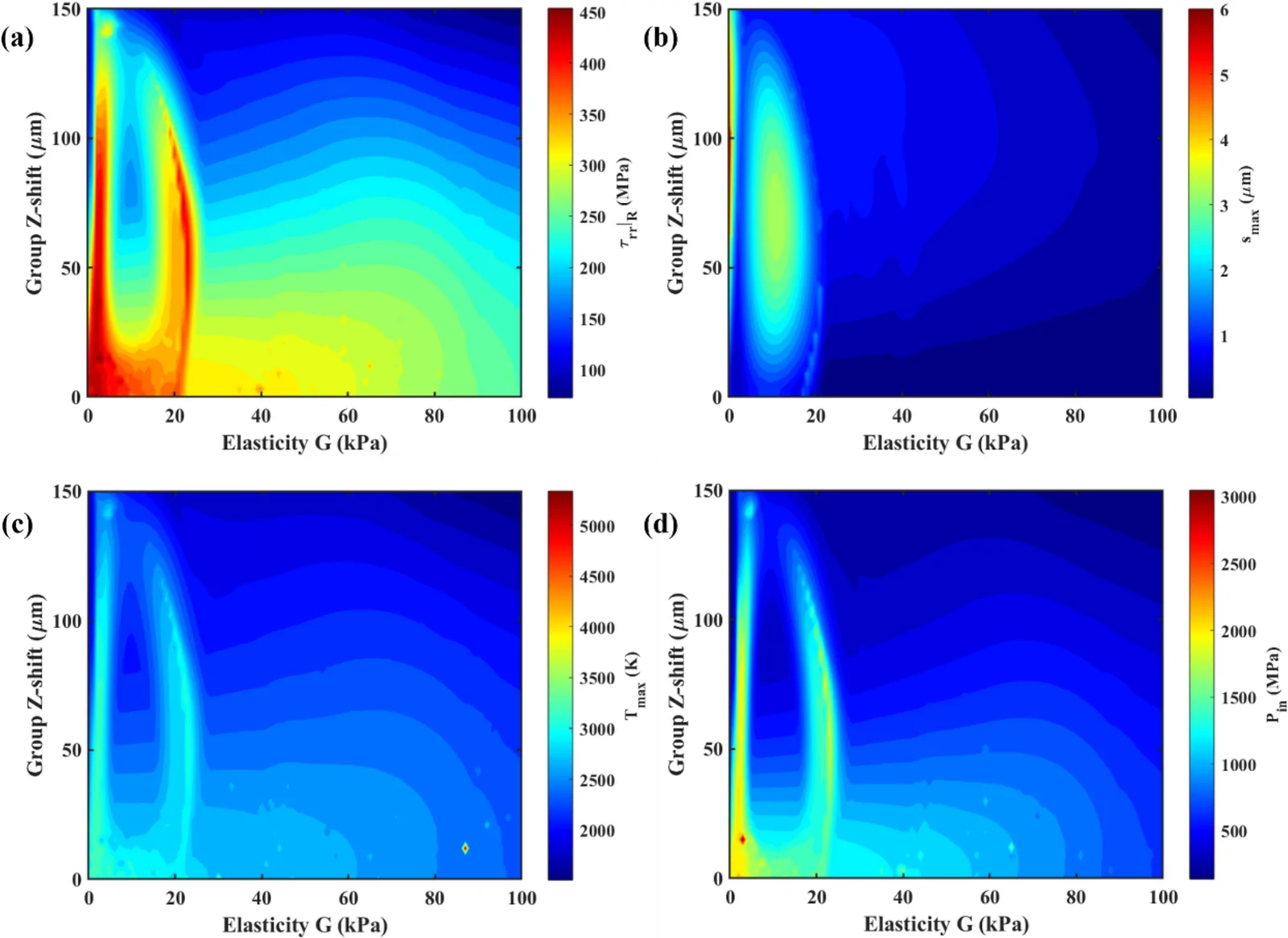
Two-dimensional contour plots illustrating the coupled effects of spatial translation and tissue elasticity. The panels display the absolute maximum values for (a) viscoelastic radial stress, (b) translational displacement, (c) internal temperature, and (d) internal pressure across the parameter space of spatial shift (0 to 150 μm) versus tissue elasticity (0 to 100 kPa). The cluster scale is maintained at a population of 10 bubbles under an acoustic driving pressure of 0.5 MPa, and the viscosity is configured to 5 mPa·s.

In contrast to the continuous gradient variations induced by tissue elasticity, viscosity introduces a pronounced damping transition. Fig. 10, Fig. 11 evaluate the parameter spaces of viscosity (0 to 100 mPa·s) against bubble count (N = 4 to 40) and spatial translation (Z = 0 to 150 μm) at a baseline tissue elasticity of 1 kPa. Both contour maps display a strict vertical boundary at a viscosity of 15 mPa·s.

**Fig. 10:**
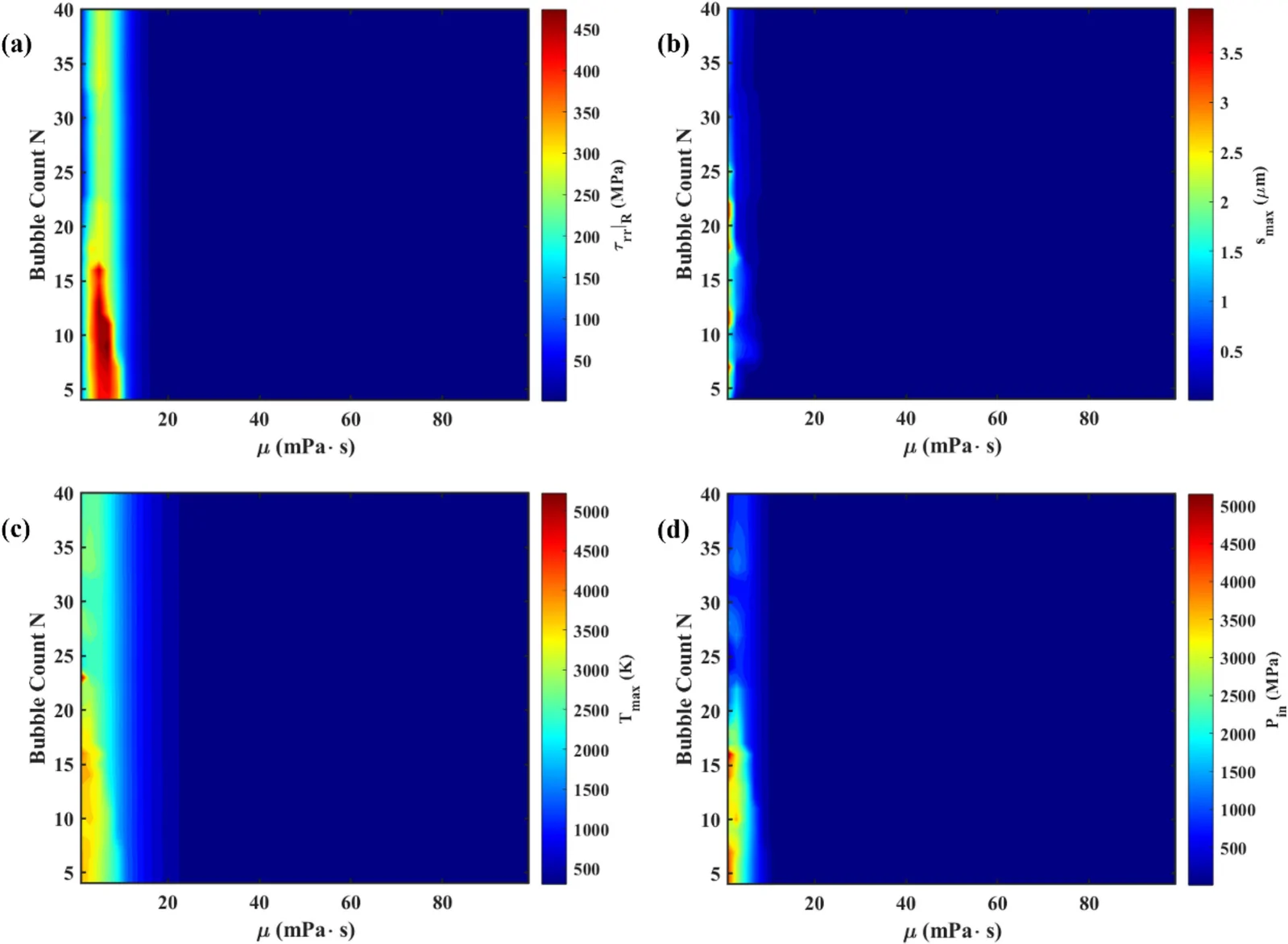
Two-dimensional contour plots evaluating the coupled effects of bubble cluster scale and viscosity. (a) viscoelastic radial stress, (b) translational displacement, (c) internal temperature, and (d) internal pressure across the parameter space of bubble count (4 to 40) versus medium viscosity (0 to 100 mPa·s). The tissue elasticity is set to 1 kPa. The spatial shift is fixed at the acoustic focal zone of 0 μm under an acoustic driving pressure of 0.5 MPa.

**Fig. 11:**
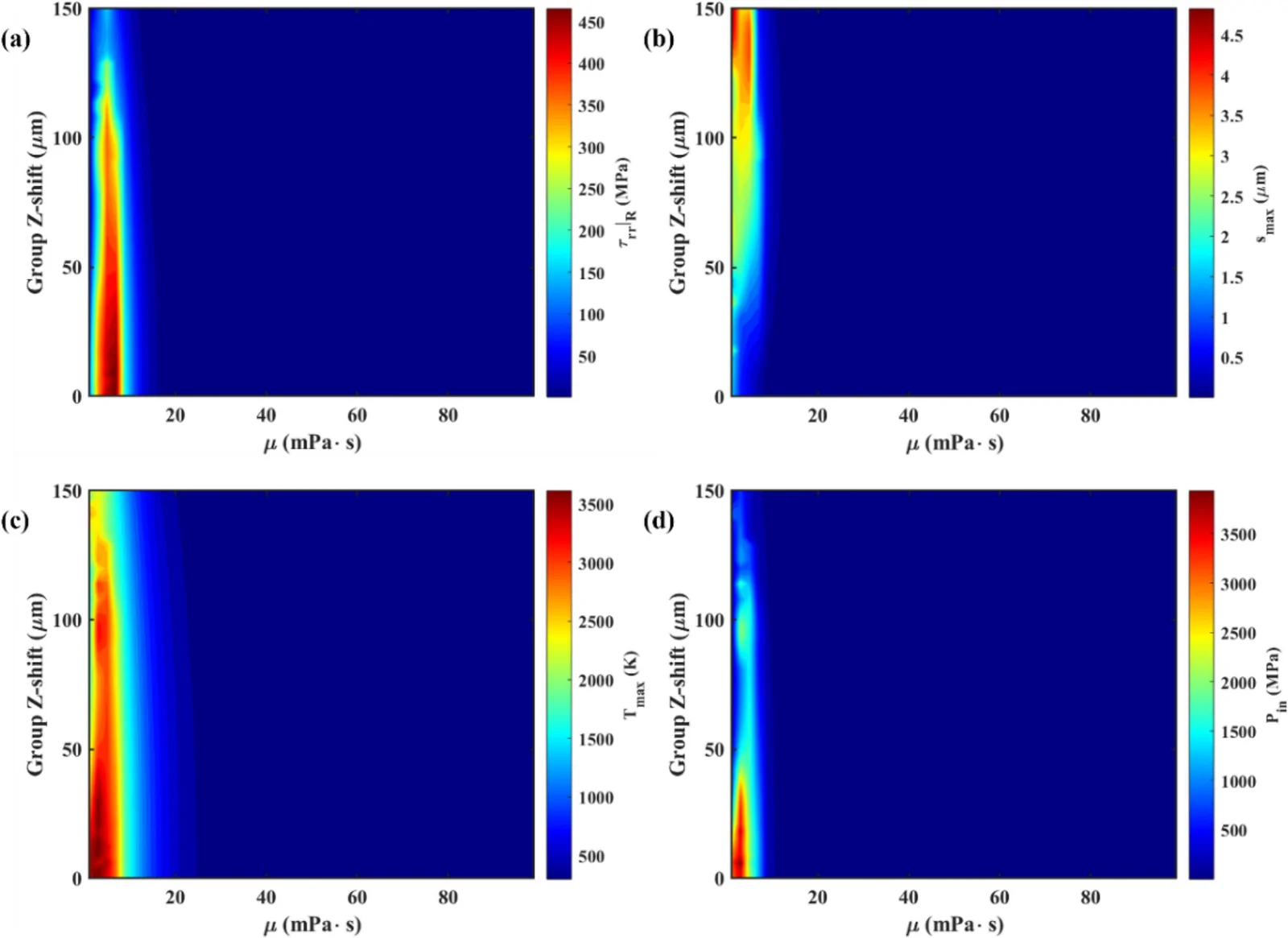
Two-dimensional contour plots demonstrating the coupled effects of spatial translation and viscosity. (a) viscoelastic radial stress, (b) translational displacement, (c) internal temperature, and (d) internal pressure across the parameter space of spatial shift (0 to 150 μm) versus medium viscosity (0 to 100 mPa·s). The cluster scale is maintained at a population of 10 bubbles, the tissue elasticity is set to 1 kPa, and the acoustic driving pressure is uniform at 0.5 MPa.

Below this viscosity threshold (10 mPa·s), the cavitation metrics reach their global maximums. Specifically, for smaller cluster scales at the focal zone, the radial stress peaks beyond 450 MPa, the internal pressure exceeds 3000 MPa, and the internal temperature approaches 5000 K. However, once the viscosity crosses the 15 mPa·s threshold, a near-total quantitative collapse occurs regardless of the topological or spatial parameters. Across the entire extended domains of N = 4 to 40 and Z = 0 to 150 μm, severe viscous dissipation flattens the output metrics: radial stress uniformly flatlines below 50 MPa, internal temperature drops below 1000 K, and translational displacement is strictly locked at 0 μm. These numerical boundaries confirm that while spatial off-centering can compensate for moderate elastic constraints up to 25 kPa, environments with dynamic viscosities exceeding 15 mPa·s strongly attenuate energetic multi-bubble cavitation.

### 3.4 Frequency tuning for 3D cluster optimization

For each response quantity, the effective resonance frequency was defined as the driving frequency at which the maximum response was obtained within the investigated frequency range. The quantities considered were the target bubble expansion ratio, radial stress, internal pressure, internal temperature, and translational displacement. Different response quantities may exhibit maxima at different frequencies. A maximum at the boundary of the investigated range does not establish the location of a resonance peak outside that range. Fig. 12 illustrates the frequency-dependent dynamics of the target bubble at driving frequencies of 0.8, 1.0, and 1.2 MHz, covering a typical therapeutic frequency range [16]. For the selected 10-bubble cluster at Z=0 μm, 1.0 MHz produces the strongest cavitation and translational response with R/R0>5, internal pressure exceeding 1600 MPa, and total displacement approaching 4.8 μm. In contrast, the responses at 0.8 and 1.2 MHz are weaker, indicating that the cluster has a frequency-sensitive response rather than a broadband maximum.

**Fig. 12:**
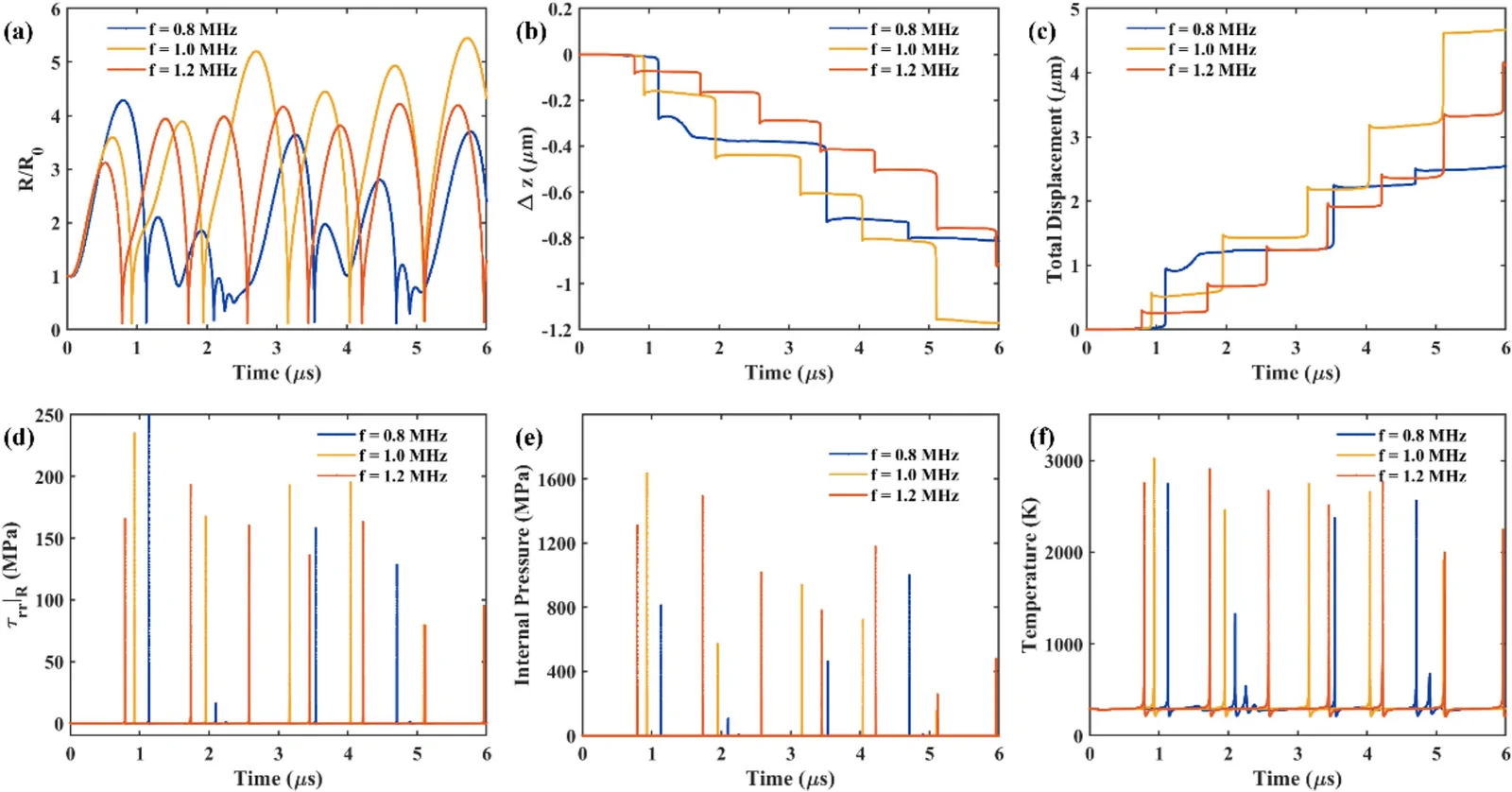
Transient dynamic profiles of the core target bubble evaluated at acoustic driving frequencies of 0.8, 1.0, and 1.2 MHz. The temporal evolution is plotted for (a) bubble expansion ratio, (b) axial displacement, (c) total displacement, (d) radial stress, (e) internal pressure, and (f) internal temperature. The fixed parameters are set to N = 10, Z = 0 μm, viscosity μ = 3 mPa·s, tissue elasticity G = 0 Pa, and an acoustic pressure of 0.5 MPa.

Crucially, the effective resonance frequency of a multi-bubble system is not static; it is inherently coupled to the cluster topology. Fig. 13 characterizes the coupled effects of bubble count and driving frequency across N=4∼40 and f=0.5∼2.0 MHz. For sparse clusters with (N<15), the high-response region for radial stress and internal temperature is mainly concentrated at 0.5–0.7 MHz. As N increases, this region becomes narrower and shifts toward lower frequencies, while translational displacement is reduced at higher frequencies [18].

**Fig. 13:**
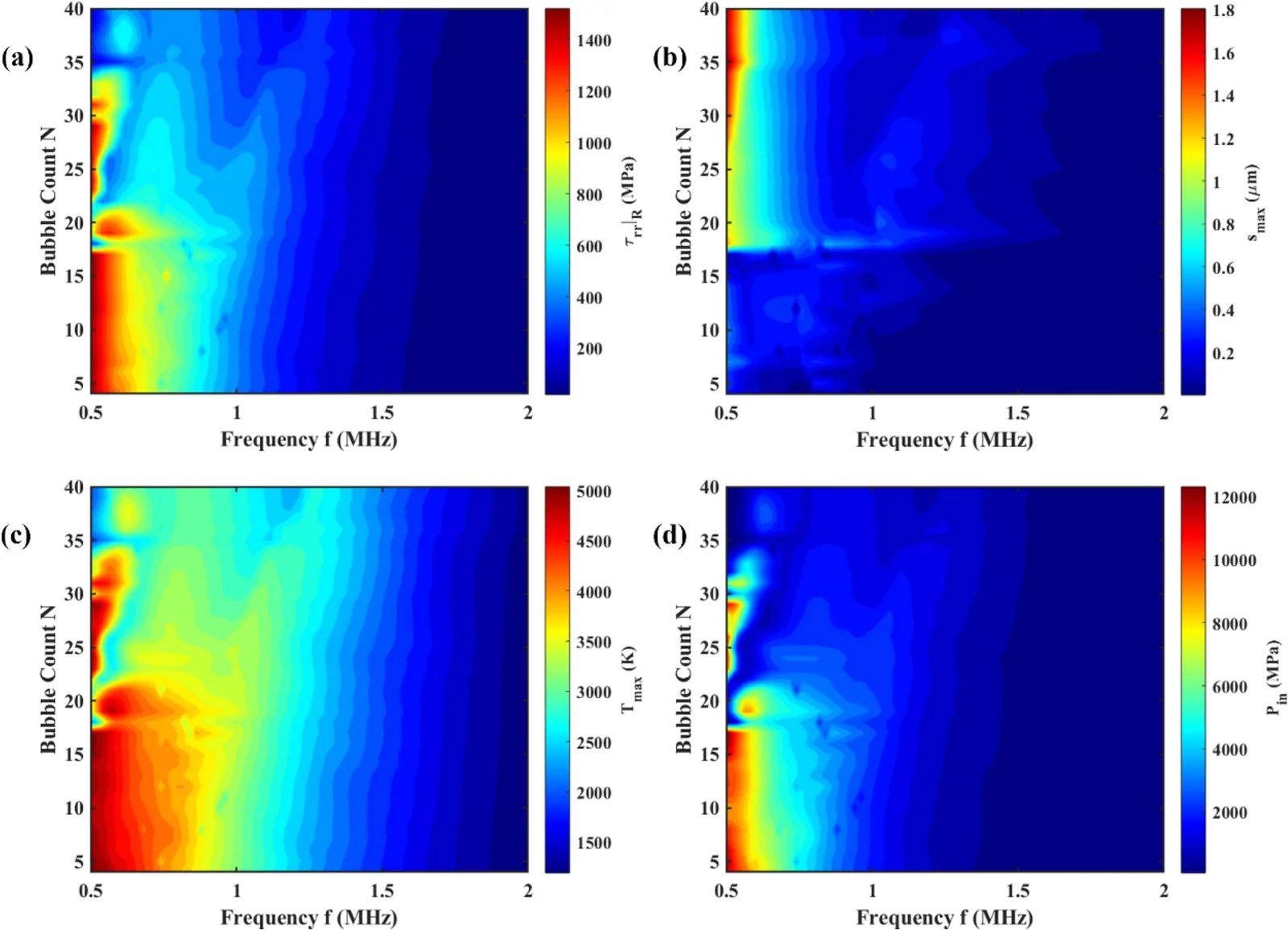
Two-dimensional contour plots illustrating the coupled effects of bubble cluster scale and driving frequency on the shifted resonance. The panels display the absolute maximum values for (a) radial stress, (b) translational displacement, (c) internal temperature, and (d) internal pressure across the parameter space of bubble count N (4 to 40) versus driving frequency f (0.5 to 2.0 MHz). The fixed soft-tissue parameters are viscosity μ = 5 mPa·s, elasticity G = 1 kPa, spatial shift Z = 0 μm, and an acoustic pressure of 0.5 MPa.

Fig. 14 further evaluates the coupled effects of spatial translation and driving frequency. Maximum internal pressure and temperature remain concentrated near the focal region and at lower frequencies of 0.5–0.7 MHz. In contrast, the peak translational displacement occurs at off-center positions of Z=80∼150 μm, where the corresponding frequency band shifts to approximately 1.0–1.5 MHz. These results indicate that the optimal frequency for thermodynamic collapse differs from that for translational migration.

**Fig. 14:**
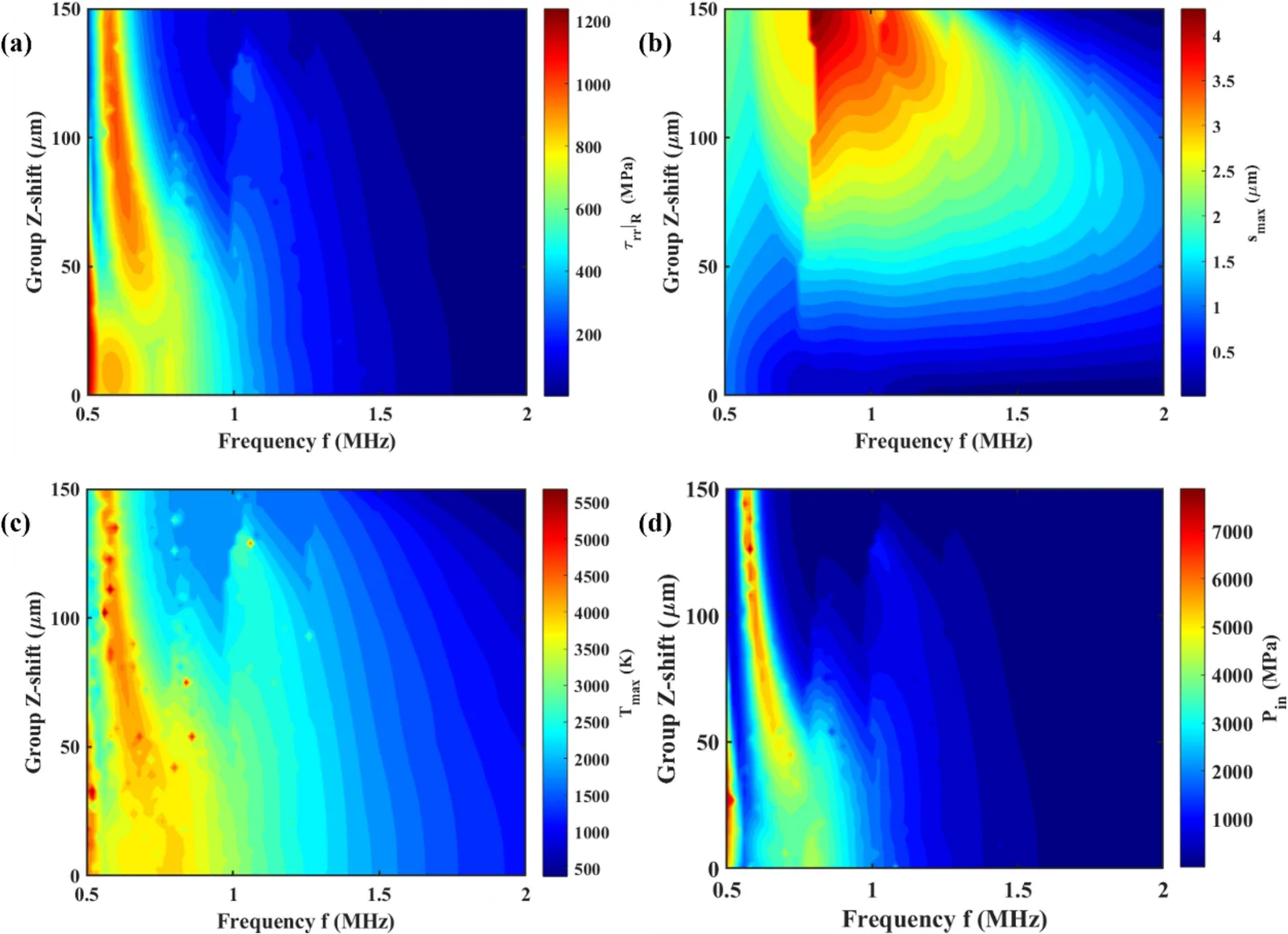
Two-dimensional contour plots demonstrating the coupled effects of spatial translation and driving frequency, highlighting the pressure-dependent resonance shift. The panels display the absolute maximum values for (a) radial stress, (b) translational displacement, (c) internal temperature, and (d) internal pressure across the parameter space of spatial shift Z (0 to 150 μm) versus driving frequency f (0.5 to 2.0 MHz). The fixed parameters are set to a cluster scale of N = 10 bubbles, viscosity μ = 5 mPa·s, elasticity G = 1 kPa, and an acoustic pressure of 0.5 MPa.

## 4 Discussion

Bubble cluster cavitation in viscoelastic tissues involves radial oscillation, gas compression, bubble–bubble coupling, and Bjerknes force driven translation, which cannot be fully described by treating each bubble as an isolated oscillator. In this study, the proposed model is applied to analyze these coupled processes in one-, two-, and three-dimensional bubble configurations. The simulations further examine how cluster size, spatial position, tissue elasticity, viscosity, and driving frequency modify the target-bubble response. Therefore, the discussion focuses on the physical implications of these coupled effects, starting from the topology-dependent collective suppression observed in 3D bubble clusters.

A fundamental mechanism revealed by this model is topology-dependent collective cavitation suppression. As the cluster scale increases, surrounding bubbles modify the local oscillation environment of the target bubble through collective interactions, thereby reducing collapse intensity across a large portion of the parameter space. This behavior may arise from altered pressure coupling among multiple bubble interfaces and the resulting modification of the local pressure field. However, this suppression is strongly dependent on spatial configuration and can be partially compensated by bubble translation. When the cluster is shifted to an intermediate off-center position, the translational displacement increases because the primary Bjerknes force becomes better phase matched with bubble volume oscillation. Thus, the strongest collapse response occurs near the acoustic focus, whereas the largest displacement appears at off-center positions, indicating that spatial positioning provides an additional control parameter for balancing cavitation intensity and translational migration in 3D bubble clusters. This spatial off-centering effect is further supported by investigations by Doinikov [46], who demonstrated that interacting acoustic bubbles can exhibit translational bouncing and self-oscillation under specific pressure thresholds.

The results further clarify the distinct roles of tissue elasticity and viscosity in regulating clustered bubble dynamics. Elasticity mainly modifies the oscillation phase and resonance condition of the bubble cluster. Although increasing elasticity generally restricts radial excursion, intermediate elastic conditions can locally enhance late-stage collapse and translational response. In contrast, viscosity acts as a dissipative constraint by removing energy from bubble oscillations. Within the examined parameter range, increasing viscosity strongly reduces radial stress, internal pressure, internal temperature, and translational displacement across different cluster sizes and spatial positions. This indicates that viscosity imposes a stronger damping limitation on energetic cavitation than tissue stiffness under the present conditions. The importance of viscoelastic boundary conditions for bubble stability and collapse behavior is also supported by recent studies on encapsulated bubbles in viscoelastic fluids [47].

Another important implication is that the effective resonance frequency of a three-dimensional bubble cluster deviates from the classical single-bubble resonance picture. Increasing cluster size shifts the high-response region toward lower frequencies, whereas spatial translation shifts the frequency associated with maximum translational displacement toward a higher band. Therefore, the optimal frequency for thermodynamic collapse is not necessarily identical to that for translational migration. This non-monotonic frequency dependence is consistent with previous findings that bubble–bubble interactions and polydispersity can alter nonlinear acoustic-wave propagation and energy dissipation in bubbly liquids [26]. These results suggest that broadband, dual-frequency, or frequency tunable excitation may be more effective than fixed single frequency excitation for controlling clustered bubbles in viscoelastic tissues.

The predicted pressure and temperature peaks may influence local sonochemical environments by affecting vapor dissociation, reaction rates, and reactive-species production. Radial stress and bubble translation may also affect the redistribution, fragmentation, and restructuring of nearby suspended particles. These possible effects provide a connection to nucleation and structural modification relevant to sonochemical nanostructuring. The present model does not directly calculate chemical species concentrations, reaction pathways, particle restructuring, or product yields, so these statements describe possible mechanistic implications rather than predictions of sonochemical yield. Several limitations should be noted. The present model assumes a homogeneous viscoelastic medium, a linearly propagating acoustic field, and spherical bubble oscillations. Nonspherical deformation, jet formation, shell effects, coalescence, fragmentation, and direct bubble contact are not considered. In addition, vascular confinement and rigid wall effects are neglected, although these boundaries may significantly affect bubble dynamics and local mechanical stresses. Future work incorporating realistic vascular geometries and experimental validation will be important for extending the present 3D cluster framework toward biological applications.

The predicted peak pressures of several gigapascals and temperatures of several thousand kelvin should be interpreted within the assumptions of the present model. Their quantitative accuracy during extreme compression depends on the applicability of the Peng–Robinson equation of state and the approximations used to describe heat and mass transfer, together with the assumptions of spherical bubble motion and a homogeneous viscoelastic medium. These results provide a basis for comparing the responses of different bubble configurations under the simulated conditions, while the absolute peak values require further validation.

## 5 Conclusion

In this study, a fully coupled three-dimensional bubble-cluster framework was developed to investigate cavitation and translational dynamics in viscoelastic tissues. By integrating the Gilmore-Akulichev formulation, the Peng-Robinson equation of state, the Jeffreys viscoelastic model, and an extended Doinikov’s translational model, the framework enables unified analysis of bubble oscillation, thermodynamic response, inter-bubble interaction, and collective migration.

The results demonstrate that bubble-cluster topology strongly regulates the cavitation response of the target bubble. Compared with the isolated GAZPREOS single-bubble baseline, the 2D and 3D cluster configurations reduce radial expansion and markedly suppress collapse-induced thermal and pressure extremes, indicating topology-dependent collective cavitation suppression. Under the present conditions, a transition in cluster response occurs near N=15–18 bubbles, beyond which the high-response cavitation region becomes progressively restricted and the cluster tends toward weaker, more stable oscillations. Spatial translation further modulates this behavior, and the optimal condition for translational migration does not necessarily coincide with that for maximum cavitation intensity. In particular, off-center positions can enhance translational displacement by improving the phase matching between bubble volume oscillation and the primary Bjerknes force.

Medium viscoelasticity produces distinct regulatory effects on multi-bubble cavitation. Tissue elasticity mainly modifies the oscillation phase and resonance condition of the cluster. Although increasing elasticity generally restricts radial excursion, intermediate elastic conditions (e.g., G=10 kPa), may induce delayed collapse amplification and localized translational spikes. In contrast, viscosity acts as a stronger dissipative constraint. Within the examined parameter range, increasing viscosity beyond approximately 15 mPa·s produces strong damping effects, substantially reducing radial stress, internal pressure, internal temperature, and translational displacement.

The effective resonance of a three-dimensional bubble cluster is also highly dependent on cluster scale and spatial position. Increasing the bubble population shifts the high-response frequency band toward lower frequencies, whereas spatial off-centering shifts the frequency for maximum translational displacement toward a higher range. These findings indicate that controlling clustered bubble dynamics requires simultaneous consideration of topology, spatial position, rheology, and driving frequency. Overall, this study provides a theoretical basis for regulating bubble-mediated mechanical effects and may contribute to the optimization of ultrasound-based therapeutic applications [11], [48], [49], [50].

## CRediT authorship contribution statement

**Zhenxiang Ji:** Validation, Investigation, Formal analysis, Conceptualization. **Zhetao Shangguan:** Visualization, Investigation. **Jie Jin:** Investigation. **Ruoyan Yang:** Data curation. **Wei Li:** Resources, Funding acquisition. **Tianyi Yan:** Supervision, Funding acquisition. **Dingjie Suo:** Writing – review & editing, Supervision, Project administration, Funding acquisition.

## Declaration of competing interest

The authors declare that they have no known competing financial interests or personal relationships that could have appeared to influence the work reported in this paper.
